# An in vitro toolbox to accelerate anti-malarial drug discovery and development

**DOI:** 10.1186/s12936-019-3075-5

**Published:** 2020-01-02

**Authors:** Susan A. Charman, Alice Andreu, Helena Barker, Scott Blundell, Anna Campbell, Michael Campbell, Gong Chen, Francis C. K. Chiu, Elly Crighton, Kasiram Katneni, Julia Morizzi, Rahul Patil, Thao Pham, Eileen Ryan, Jessica Saunders, David M. Shackleford, Karen L. White, Lisa Almond, Maurice Dickins, Dennis A. Smith, Joerg J. Moehrle, Jeremy N. Burrows, Nada Abla

**Affiliations:** 1 Centre for Drug Candidate Optimisation, Monash Institute of Pharmaceutical Sciences, Monash University, 381 Royal Parade, Parkville, VIC 3052 Australia; 2 Certara UK Limited, Simcyp Division, Level 2-Acero, 1 Concourse Way, Sheffield, S1 2BJ UK; 3 4 The Maltings, Walmer, Kent, CT14 7AR UK; 4 Medicines for Malaria Venture, PO Box 1826, 20 Route de Pré-Bois, CH-1215 Geneva 15, Switzerland

**Keywords:** Physiologically-based pharmacokinetic modelling, Anti-malarial drugs, Ionization constant, Partition coefficient, Biorelevant solubility, Protein binding, Blood to plasma partitioning, Microsomal stability, CYP inhibition

## Abstract

**Background:**

Modelling and simulation are being increasingly utilized to support the discovery and development of new anti-malarial drugs. These approaches require reliable in vitro data for physicochemical properties, permeability, binding, intrinsic clearance and cytochrome P450 inhibition. This work was conducted to generate an in vitro data toolbox using standardized methods for a set of 45 anti-malarial drugs and to assess changes in physicochemical properties in relation to changing target product and candidate profiles.

**Methods:**

Ionization constants were determined by potentiometric titration and partition coefficients were measured using a shake-flask method. Solubility was assessed in biorelevant media and permeability coefficients and efflux ratios were determined using Caco-2 cell monolayers. Binding to plasma and media proteins was measured using either ultracentrifugation or rapid equilibrium dialysis. Metabolic stability and cytochrome P450 inhibition were assessed using human liver microsomes. Sample analysis was conducted by LC–MS/MS.

**Results:**

Both solubility and fraction unbound decreased, and permeability and unbound intrinsic clearance increased, with increasing Log D_7.4_. In general, development compounds were somewhat more lipophilic than legacy drugs. For many compounds, permeability and protein binding were challenging to assess and both required the use of experimental conditions that minimized the impact of non-specific binding. Intrinsic clearance in human liver microsomes was varied across the data set and several compounds exhibited no measurable substrate loss under the conditions used. Inhibition of cytochrome P450 enzymes was minimal for most compounds.

**Conclusions:**

This is the first data set to describe in vitro properties for 45 legacy and development anti-malarial drugs. The studies identified several practical methodological issues common to many of the more lipophilic compounds and highlighted areas which require more work to customize experimental conditions for compounds being designed to meet the new target product profiles. The dataset will be a valuable tool for malaria researchers aiming to develop PBPK models for the prediction of human PK properties and/or drug–drug interactions. Furthermore, generation of this comprehensive data set within a single laboratory allows direct comparison of properties across a large dataset and evaluation of changing property trends that have occurred over time with changing target product and candidate profiles.

## Background

The number of deaths due to malaria has dropped substantially in recent years, from more than 800,000 in 2000 [1] to approximately 435,000 in 2017 [2]. This reduction has been attributed in large part to the widespread use of artemisinin-based combination therapy (ACT) and insecticide-treated bed nets as well as improved vector control. However, the most recent estimates from the World Health Organization (WHO) suggest that the malaria incidence rate per 1000 population at risk has been steady at 59 for the past 3 years suggesting that progress in reducing infection has reached a standstill [2]. The factors contributing to these trends are many, including parasite resistance to existing drugs, mosquito resistance to insecticides, lack of sustained and predictable financing for malaria eradication programmes in disease endemic countries, poor performance of regional health systems and various regional conflicts [3].

Since 2000, there has been a considerable increase in anti-malarial drug discovery leading to a relatively healthy pipeline of promising new drug candidates in preclinical and clinical development [4]. Over this same time period, new drug approvals have included new artemisinin-based combinations, new combinations of other existing drugs, and new and improved formulations, each of which has contributed significantly to the anti-malarial arsenal. However there have been only two new drug approvals containing new chemical entities (Synriam, a combination of the novel ozonide arterolane or OZ277 and piperaquine, and Krintafel/Kozenis containing tafenoquine) over this same period, and of these, only tafenoquine has undergone stringent regulatory approval by International Conference on Harmonization (ICH) members or observers. This scenario reflects the relatively limited emphasis on anti-malarial drug discovery prior to about 2000, and the inevitable timeframe required to progress new compounds through discovery, translational and clinical development. The situation is further exacerbated by the need for combination therapies, preferably delivered in a single dose, to treat all parasitic forms and reduce the development of drug resistance, and the associated complexity of obtaining efficacy, safety, and pharmacokinetic data for individual agents before they are combined.

Given this landscape, it is essential that improved methods to accelerate the discovery and development of malaria drugs are implemented so that new and more convenient medicines can be made available to patients in a shorter period of time. Modelling and simulation tools have received considerable attention in recent years, are well established in the industry [5–10] and are being increasingly recognized by regulatory authorities [11–15]. These approaches are now also being applied in the discovery and development of anti-malarial drugs as recently reviewed by Andrews et al. [16]. The availability of improved preclinical models for assessing efficacy against human parasitic infections [17], as well as the establishment of volunteer infection studies (VIS) [18–21], has reduced the time required to establish preclinical and clinical proof of concept and provides a rich supply of data for the development of pharmacokinetic/pharmacodynamic models [22–25].

Fundamental to many of these modelling initiatives is the use of physiologically-based pharmacokinetic (PBPK) modelling. This predictive tool is a mechanistic whole-body distribution model that incorporates compound specific data (e.g. physicochemical, permeability, binding and clearance) along with physiological (e.g. tissue composition, volume and organ blood flow) and population specific data to simulate absorption, distribution and elimination profiles. As highlighted in a recent white paper, these methods are being increasingly recognized by the FDA for first-in-human dose selection and to predict clinical drug–drug interactions [6, 14, 26–28]. Previous reports have highlighted the need for reliable compound specific data to improve the predictability of PBPK models [6]. While there are numerous in silico methods available for predicting physicochemical properties, there are still inherent flaws in being able to accurately predict certain parameters that impact the outcome of PBPK predictions.

The current work was undertaken as part of a broad collaboration between the Medicines for Malaria Venture, the Bill & Melinda Gates Foundation and Simcyp (Certara UK Limited) to demonstrate the utility of PBPK modelling and simulation to accelerate the discovery and development of fixed-dose combinations for new anti-malarial drugs. The first stage of the project, which is the subject of this manuscript, was to generate in vitro data to support PBPK modelling, including physicochemical, permeability and binding properties, intrinsic clearance, and cytochrome P450 inhibition constants for a set of legacy anti-malarial drugs and drug candidates in preclinical and clinical development using standardized conditions. The second stage, which will be published separately, was to use the data set to build PBPK models for legacy compounds and make them available to malaria researchers. These models will be used for different applications such as simulations of drug–drug interactions of new combinations containing legacy compounds. The final stage, which is still on-going, is to implement the PBPK methodology into candidate selection and clinical development of new anti-malarial drug combinations. This manuscript reports the in vitro data set generated for a total of 45 compounds of which 23 are legacy drugs, 2 are active metabolites, and 20 are preclinical and clinical development compounds (including 2 recently introduced new drugs) with details of the methodology used to obtain these data.

## Methods

### Materials

All compounds were obtained from the Medicines for Malaria Venture, Geneva, Switzerland. Structures, salt forms and current development status for all compounds in the data set are shown in Additional file 1: Table S1. The data set includes 20 development compounds that are either in preclinical or clinical development or have been recently approved (OZ277 or Arterolane and tafenoquine), 23 legacy compounds that are currently used clinically or have been used in the past, and two active metabolites (desethylamodiaquine and cycloguanil). The launched drug list was obtained from the public database ChEMBL (https://www.ebi.ac.uk/chembl/) and included 274 oral drugs launched between 2000 and 2017 excluding enzymes, oligopeptides, polymers, buffering agents, and amino acids, and drugs that have been withdrawn or discontinued.

### Molecular property descriptors

Molecular property descriptors were calculated using ChemAxon JChem for Excel version 18.5.0.196 (ChemAxon, Budapest, Hungary). For the ChEMBL oral drug set, SMILES strings were used to calculate the molecular property descriptors using ChemAxon.

### Instrumentation and sample analysis

Sample analysis was conducted by LC–MS/MS using a Waters Acquity UPLC system (Waters Corporation, Milford, MA) coupled to either a triple quadrupole mass spectrometer (Waters Micromass Quattro Premier, Waters Micromass Quattro Ultima PT, Waters Xevo TQ, or Waters Xevo TQD) for quantitative analysis or a time of flight mass spectrometer (Waters Xevo G2 QToF) for the assessment of metabolism. For samples where concentrations were high (e.g. some of the partitioning and solubility samples), detection was conducted by UV absorption rather than MS/MS. Details of the sample preparation procedures are provided within each of the individual method sections. In all cases, quantitation was conducted by comparison of the sample response (peak area ratio using diazepam as an internal standard) to the response for a set of calibration standards prepared in the same matrix, bracketing the expected concentration range and analysed at the same time as the study samples. Representative analytical conditions are shown in Additional file 1: Table S2 with typical validation data shown in Additional file 1: Table S3.

### Ionization constants

Ionization constants were calculated using in silico methods and measured experimentally. In silico methods included the ADMET Predictor module embedded within the PBPK software package, GastroPlus, ver. 9.6 (Simulations Plus, Inc, Lancaster, CA) and ChemAxon JChem for Excel. Calculated values from the public database ChEMBL (https://www.ebi.ac.uk/chembl/, ACD Labs ver. 12.01) were included for compounds available within the ChEMBL database.

Ionization constants were measured by potentiometric titration using a Metrohm 809 Titrando autotitrator (Metrohm AG, Switzerland) equipped with an 800 Dosino burette (2 mL), an 800 stirring unit and a jacketed reaction vessel capable of titrating volumes between 2 and 10 mL. The autotitrator was controlled by Tiamo software (Version 1.3). pH measurements were conducted using a Metrohm LL Micro glass electrode which was calibrated on the day of use with calibration standards at pH 2, 4, 7 and 10. All reagents were standardized (directly or indirectly) against potassium hydrogen phthalate (Sigma-Aldrich, A.C.S. Acidimetric Standard). Titrant solutions were protected from carbon dioxide absorption by flushing with nitrogen before sealing or by the incorporation of a drying tube filled with self-indicating soda lime into the titration reaction vessel set-up.

A stock solution of each compound was prepared in DMSO typically at a concentration of 5 mM. Aliquots were introduced directly into the titration vessel and diluted 1:10 with water (typical final compound concentration of 0.5 mM). Titrations were performed in triplicate with standardized hydrochloric acid or potassium hydroxide (10 mM) and titrant volume increments of 1 µL, resulting in a minimum of 100 data points for each titration. pKa values were obtained by fitting the data to the Henderson–Hasselbalch equation [29] and averaging the results about the 0.5 equivalent point (first pKa) and the 1.5 equivalent point (second pKa where present) of the titration.

### Partition coefficients

Partition coefficients were calculated using in silico methods and measured experimentally and. In silico methods included ADMET Predictor and ChemAxon. Calculated values from the public database ChEMBL (https://www.ebi.ac.uk/chembl/, ACD Labs ver. 12.01) were included for compounds available in the ChEMBL database.

Partition coefficients between octanol and pH 7.4 buffer were measured using a shake flask method. A stock solution of test compound in octanol was prepared at a concentration between 3 and 30 mg/mL based on the expected partition coefficient value. This stock solution was then diluted 3- and 10-fold with octanol and used to prepare the octanol phase for the partitioning experiments. Two different dilutions were used to confirm that there were no saturation effects. Phosphate buffered saline was prepared by combining 67 mM disodium hydrogen orthophosphate and sodium dihydrogen orthophosphate (both prepared in 43 mM NaCl) to a final pH of 7.4.

Partitioning experiments were conducted by mixing equal volumes of the octanol (containing test compound) and aqueous phases and placing on a vibrating plate mixer in an incubator at 37 °C. At 24 and 48 h, the samples were centrifuged (10,000 rpm × 3 min) and duplicate aliquots of the octanol phase removed and diluted first with isopropanol (1:9) and then with 50–80% aqueous methanol depending on the compound properties. An aliquot of the aqueous phase was carefully removed and centrifuged again to ensure no contamination from the octanol phase before sampling in duplicate and diluting with aqueous methanol for analysis. Diluted samples were analysed by LC–MS along with calibration standards (Additional file 1: Table S2) and partition coefficients were calculated from the ratio of the mean octanol to buffer concentration after accounting for the dilution factors. The partitioning results for the two time points were used to confirm that the partitioning experiment had reached equilibrium.

### Solubility in biorelevant media

Solubility of each active pharmaceutical ingredient was evaluated at 37 °C in pH 7.4 phosphate buffered saline (prepared as described for the partitioning experiments), fasted (FaSSIF-V2) and fed (FeSSIF-V2) state simulated intestinal fluids and fasted state simulated gastric fluid (FaSSGF) as described by Jantratid et al. [30]. Compounds were accurately weighed into individual screw cap polypropylene tubes and media added to give a nominal target compound concentration of 2 mg/mL (maximum concentration tested for most compounds). Samples were vortexed and placed in a 37 °C incubator on an orbital mixer (IKA^®^ VXR basic Vibrax^®^ orbital mixer) set at 600 rpm. Sampling times were 1 h for FaSSGF or 5–6 h for FaSSIF-V2, FeSSIF-V2, and PBS. These times were used to reflect the maximal likely residence times within the stomach and small intestine, respectively. Sampling was carried out by centrifuging each sample at 10,000 rpm for 3 min, transferring 300 µL aliquots into fresh Eppendorf tubes and centrifuging these tubes again at 10,000 rpm for 3 min. Triplicate aliquots of the supernatant were then removed and diluted 1:2 in 50% aqueous methanol and then again in 50% aqueous acetonitrile to be within the analytical concentration range. Samples were analysed by LC–MS along with calibration standards (Additional file 1: Table S2).

### Permeability

Bidirectional permeability was assessed across Caco-2 cell monolayers as described previously [31]. Briefly, permeability experiments were performed using either aqueous transport buffer (pH 7.4 Hanks balanced salt solution containing 20 mM HEPES) or human plasma (Australian Red Cross Blood Service) in both the apical and basolateral chambers. Donor solutions were prepared by spiking stock solutions into transport media to give a final compound concentration in the range of 10–20 µM (using buffer as the transport medium) or 10–50 µM (using plasma as the transport medium; note that the unbound donor concentration will vary depending on the fraction unbound). The final DMSO concentration in the donor solution was 0.1% v/v. Donor solutions were equilibrated at 37 °C for up to 4 h before centrifuging at 4000 rpm for 5 min to remove any compound that may have precipitated.

Compound flux was assessed over a maximum period of 90–180 min, with samples taken periodically from the acceptor chamber. Samples from the donor chamber were taken at the start and end of the experiment. Donor and acceptor samples for lucifer yellow and rhodamine 123 were analysed by fluorescence (FLUOstar OPTIMA plate reader; BMG Lab Technologies, Offenburg, Germany) with the excitation/emission wavelengths set at 430/535 nm for lucifer yellow and 500/525 nm for rhodamine 123. Donor and acceptor samples were stored frozen at − 80 °C until analysis by LC–MS (Additional file 1: Table S2) with sample preparation as described previously [31]. The mass balance and apparent permeability coefficient (P_app_) were calculated as previously described [31].

Where human plasma was used as the transport medium, P_app_ values were calculated as shown above, with correction for the fraction unbound (f_u_) in the donor solution $\left( {{\text{C}}_{\text{donor}}^{\text{initial}} \times {\text{f}}_{\text{u}} } \right)$ with f_u_ determined at a similar concentration to that used in the transport experiment. The apparent flux of lucifer yellow was based on an endpoint measurement assuming no lag time. The efflux ratio was calculated as the ratio of the mean B–A to A–B P_app_ values.

### Solubility limited absorbable dose calculations

The solubility limited absorbable dose (SLAD) was calculated as previously described [32] using Eq. (1):

1 $${\text{SLAD }} = {\text{ S}}_{\text{si}} \times {\text{V }} \times {\text{ M}}_{\text{p}}$$

where S_si_ is the estimated solubility in the small intestine (based on the measured FaSSIF solubility), V is the fluid volume (500 mL), M_p_ is the permeability multiplier (equivalent to the absorption number (A_n_ = P_eff_ × t_res_/R, where P_eff_ is the predicted effective human jejunal permeability, t_res_ is the mean residence time in the small intestine (3.32 h [32]), and R is the radius of the small intestine (1 cm) [33])) with a minimum value of 1 for poorly permeable compounds. Predicted P_eff_ values were obtained from a calibration plot of literature P_eff_ values [34, 35] vs measured Caco-2 P_app_ [31] using either buffer or plasma as the transport medium (see Results section). The maximum value for Caco-2 P_app_ was conservatively taken to be 3 × 10^−4^ cm/s giving a maximum value for P_eff_ of ~ 1 × 10^−3^ cm/s which is consistent with previous reports [33, 35].

### In vitro protein binding

#### Media sources

Pooled human plasma (n = 3–4 donors) was obtained by centrifugation of blood (collected by the Australian Red Cross Blood Service, Melbourne, Australia or the Volunteer Blood Donor Registry, Clinical Translation Centre, Walter & Eliza Hall Institute of Medical Research, Parkville, Australia), or sourcing pooled plasma directly from commercial sources (Innovative Research Inc, MI) and stored frozen at − 80 °C. On the day of the experiment, frozen plasma was thawed and either neat or diluted plasma aliquots were spiked with a compound stock solution (prepared in 20/40/40 (v/v) DMSO/acetonitrile/water) to give a final nominal concentration of 1000–2000 ng/mL and maximum final DMSO and acetonitrile concentrations of 0.2% (v/v) and 0.4% (v/v), respectively.

A suspension of human liver microsomes (HLM, XenoTech LLC, Lenexa, KS, USA) was prepared in 0.1 M phosphate buffer (pH 7.4) at a protein concentration of 0.4 mg/mL immediately prior to the experiment. An aliquot of the HLM matrix was spiked with compound stock solution as described above to give a final concentration of 0.5–1 µM with final DMSO and acetonitrile concentrations of 0.004% (v/v) and 0.1% (v/v), respectively.

Albumax medium was prepared as per the manufacturer’s instructions and contained Albumax II (lipid rich bovine serum albumin; 5.0 g/L, Gibco, Thermo Fisher Scientific), RPMI 1640 powder (Gibco; 1 sachet or 10.4 g/L; contains l-glutamine 0.3 g/L and sodium bicarbonate 2.1 g/L), HEPES (5.94 g/L) and neomycin (100 mg/L). An aliquot of Albumax medium was spiked with a compound stock solution as described above to give a final concentration of 500 ng/mL with final DMSO and acetonitrile concentrations of 0.2% (v/v) and 0.4% (v/v), respectively.

Dulbecco’s Modified Eagle’s Medium (DMEM) containing GlutaMAX-I was purchased from Invitrogen (Thermo Fisher Scientific) and stored at 4 °C. Medium was prepared by adding heat inactivated foetal calf serum (FCS, final 10% v/v), penicillin (final 100 U/mL), streptomycin (final 100 µg/mL) d-glucose (final 4.0 mg/mL) and sodium pyruvate (final 0.1 mg/mL). Aliquots of medium were spiked with compound stock solutions as described above to give a final concentration of 1000 ng/mL and maximum final DMSO and acetonitrile concentrations of 0.2% (v/v) and 0.4% (v/v), respectively.

#### Protein binding via ultracentrifugation (UC)

An ultracentrifugation method adapted from a previous publication [36] was initially used to assess plasma protein binding and binding in the other media. Spiked plasma, Albumax or DMEM/FCS medium was vortex mixed briefly and aliquots (n = 3–4) transferred to ultracentrifuge tubes which were allowed to equilibrate for 30–45 min at 37 °C in an atmosphere of 5% (for plasma or Albumax) or 10% (for DMEM/FCS) CO_2_ before being transferred to a rotor (Beckman Rotor type 42.2 Ti; 223,000×*g*). The rotor was maintained for a further 15 min under the same CO_2_ atmosphere and the pH was confirmed to be within pH 7.4 ± 0.1 before the rotor was sealed and subjected to ultracentrifugation at 37 °C for 4.2 h. For microsomes, samples were equilibrated for 30–45 min at 37 °C under ambient atmosphere since microsomes are suspended in phosphate buffer and, therefore, not subject to the same pH shifts as for the other bicarbonate buffered media and plasma. Additional ultracentrifuge tubes containing spiked matrix were maintained at 37 °C, 5% or 10% CO_2_ or normal atmosphere conditions, with aliquots being taken within 0.5 h of the start and at the end of ultracentrifugation to serve as controls for the assessment of stability and to obtain a measure of the total concentration (C_total_). Following ultracentrifugation, the pH was checked and an aliquot of protein-free supernate was taken from each ultracentrifuge tube for determination of the unbound concentration (C_unbound_).

Total matrix and protein free samples were analysed using a matrix matching approach [37] whereby each sample was mixed in a 1:1 ratio with the opposite blank medium (i.e. blank total matrix or blank protein free buffer). For example, plasma samples were mixed with blank pH 7.4 buffer whereas plasma supernatant samples were mixed with blank plasma. Each of the sample sets were then assayed against a common calibration curve prepared in a 1:1 mixture of total matrix and protein free pH 7.4 buffer. All samples were stored at − 80 °C until analysis by LC–MS (Additional file 1: Table S2). The unbound fraction in plasma or medium was calculated using the average values for C_total_ and C_unbound_ (n = 3–4 for each). The standard deviation for f_u_ was calculated using the propagation of errors approach as described previously [38]. The potential for compound degradation was assessed by comparing the average value for C_total_ at the start and end of the experiment.

#### Protein binding via rapid equilibrium dialysis (RED)

For compounds that were found to have lower f_u_ values (nominally f_u_ < 0.1) by ultracentrifugation, binding was further assessed by RED using diluted plasma. Plasma was diluted 1:10 with pH 7.4 phosphate buffer (prepared by mixing 0.1 M sodium dihydrogen phosphate and 0.1 M disodium hydrogen phosphate (both containing 0.04 M NaCl) to pH 7.4) and spiked with compound to achieve a total measured concentration of ~ 1000–3000 ng/mL. Diluted plasma was vortex mixed briefly and aliquots (n = 3–4) were transferred to RED (Thermo Fisher Scientific, Waltham, MA) units that were placed at 37 °C under ambient atmosphere on a plate shaker. The pH of the diluted plasma was confirmed to be within pH 7.4 ± 0.1, and dialysis was conducted for a period of 6 or 24 h (see further details for the 24 h conditions below). At the end of the dialysis period, samples were removed from both the donor and dialysate chambers of the RED units. Validation experiments confirmed that the pH of 10% plasma and dialysate at the end of the experiment were each within 7.4 ± 0.1. Samples were matrix matched as described above and stored at − 80 °C until analysis by LC–MS (Additional file 1: Table S2). Stability was confirmed as for the UC assay. Fraction unbound values were calculated for each individual RED unit and the mean and SD calculated for n = 3–4 replicates.

For compounds that were very highly bound (f_u_ < 0.01) in plasma and highly lipophilic (Log D ≥ 3.5) with the potential for loss due to adsorption to the dialysis units and slow equilibration, additional measures were incorporated to ensure that the system was at steady state [39, 40]. These measures included (i) incorporating a presaturation period to saturate non-specific binding sites on the RED chamber and dialysis membrane prior to dialysis, (ii) adding unbound compound to the dialysate chamber at the start of the dialysis period to accelerate the attainment of steady state, and (iii) using a 24 h dialysis period. Briefly, the RED device was exposed for two 30 min periods and one overnight period to fresh solutions of compound prepared in pH 7.4 buffer at approximately 10% of the total dialysis concentration. Following the preincubations, solutions were removed from the RED device and discarded. To initiate the dialysis, spiked diluted plasma was added to the donor chamber and pH 7.4 phosphate buffer spiked with compound (at 1–2% of the total diluted plasma concentration) was added to dialysate chamber and dialysis allowed to proceed for 24 h at 37 °C under ambient atmosphere on a plate shaker. Samples were removed and analysed as described above.

For binding assessments using 10% plasma, the unbound fraction (f_u_) in neat plasma was calculated using the average values for C_total_ and C_unbound_ and Eq. (2), where D is the dilution factor [41]:

2 $$f_{u} = \frac{{1/{\text{D}}}}{{\left( {\left( {\frac{{{\text{C}}_{\text{total}} }}{{{\text{C}}_{\text{unbound}} }}} \right) - 1} \right) + 1/{\text{D}}}}$$

### Blood to plasma partitioning

Human whole blood was collected and supplied by the Volunteer Blood Donor Registry (Clinical Translation Centre, Walter & Eliza Hall Institute of Medical Research, Parkville, Australia) and used on the day of collection. The haematocrit (Hct) was determined by centrifugation (13,000×*g* for 3 min using Clemets^®^ Microhaematocrit centrifuge and Safecap^®^ Plain Self-sealing Mylar wrapped capillary tubes) to ensure it was between 0.40 and 0.48. An aliquot was centrifuged (Heraeus, Multifuge 3 S-R; 4500×*g*) for 10 min to obtain plasma required for matrix matching purposes as described below.

Aliquots of whole blood were spiked with compound stock solutions (prepared in 20/40/40 (v/v) DMSO/acetonitrile/water) to give a final nominal concentration of 1000 ng/mL with final DMSO and acetonitrile concentrations of 0.2% (v/v) and 0.4% (v/v), respectively. Two aliquots of the spiked whole blood were transferred to fresh microcentrifuge tubes and maintained at 37 °C/5% CO_2_ in a humidified incubator. The pH was confirmed to be 7.4 ± 0.1 at the start and end of the incubation. At each time point (30 min and 4 h), one whole blood tube was removed from the incubator and mixed by gentle inversion, after which four replicate blood samples were taken and matrix matched with an equal volume of blank plasma. The remainder of the blood sample was centrifuged (Eppendorf, Mini Spin plus; 6700×*g*) for 2 min for the collection of 4 replicate plasma samples which were similarly matrix matched with an equal volume of blank whole blood. The 1:1 mixtures of blood/plasma were mixed, snap frozen in dry ice and stored at − 80 °C until analysis by LC–MS (Additional file 1: Table S2) against calibration standards prepared in the same mixed matrix. Any further distribution of compound into RBCs at this stage was irrelevant as the cells were lysed during the sample preparation and the total concentration in the mixed matrix was measured for both the calibration standards and samples.

Compound stability in whole blood was assessed by comparing the compound concentrations measured at 30 and 240 min. The apparent whole blood-to-plasma partitioning ratio (B/P) was calculated as the ratio of the average concentration in the blood sample to that in the plasma fraction of the same whole blood sample. A standard deviation (SD) for each B/P value was calculated using the propagation of errors approach as described previously [38].

### In vitro metabolism in human liver microsomes

The metabolic stability assay was adapted from a previously published method [42]. Test compound spiking solutions (prepared in 5/95 DMSO/acetonitrile) were added to in duplicate to a suspension of human liver microsomes (0.4–0.5 mg/mL) prepared in 0.1 M phosphate buffer (pH 7.4) containing 1 U/mL glucose-6-phosphate dehydrogenase to give a final concentration of 1 µM for all compounds except JPC3210 and MMV052 which were run at 0.5 µM. Mixtures were equilibrated briefly (~ 5–10 min) at 37 °C. The metabolic reaction was initiated by the addition of an NADPH-regenerating system to give final concentrations of 1.3 mM NADP, 3.5 mM glucose-6-phosphate, and 3.3 mM MgCl_2_. Reactions were quenched at 2, 5, 15, 30 and 60 min by the addition of acetonitrile containing 150 ng/mL diazepam as internal standard. Control samples (containing no cofactor) were included (quenched at 2, 30 and 60 min) to monitor degradation in the absence of cofactor. Concentrations were determined by LC–MS (Additional file 1: Table S2) by comparison to the response for a single point calibration standard prepared in quenched microsomal matrix.

Test compound concentration versus time data were fit using an exponential decay function to determine the first-order rate constant for substrate depletion. Where deviation from first-order kinetics was evident, only the initial linear portion of the logarithmic profile was utilized to determine the initial degradation rate constant (k, min^−1^). Each substrate depletion rate constant was then used to calculate the in vitro intrinsic clearance value (CL_int_, in vitro, µL/min/mg protein) using Eq. (3).

3 $${\text{CL}}_{{{\text{int}},}} \;{\text{in}}\;{\text{vitro}} = {\text{ k }}\left( {{ \hbox{min} }^{ - 1} } \right) \, \times \, {{ 1000 \, ({{\upmu{\text{L}}} \mathord{\left/ {\vphantom {{\upmu{\text{L}}} {\text{mL}}}} \right. \kern-0pt} {\text{mL}}}}) \mathord{\left/ {\vphantom {{ 1000 \, {{\upmu{\text{L}}} \mathord{\left/ {\vphantom {{\upmu{\text{L}}} {\text{mL}}}} \right. \kern-0pt} {\text{mL}}}} {{\text{protein concentration }}\left( {{\text{mg}}/{\text{mL}}} \right)}}} \right. \kern-0pt} {{\text{protein concentration }}\left( {{\text{mg}}/{\text{mL}}} \right)}}$$

The limit of sensitivity of this assay was considered to be 15% loss of substrate over the assay duration. For compounds showing < 15% loss over 60 min, intrinsic clearance is quoted as < 7 µL/min/mg protein. Unbound in vitro CL_int_ values were obtained by dividing the measured CL_int_ by the measured f_u_ in microsomes.

### Cytochrome P450 inhibition

The CYP inhibition assay was based on a previous publication with minor modifications [43]. The method uses human liver microsomes and a substrate-specific interaction approach which relies on the formation of a metabolite that is mediated by a specific CYP isoform. The specific CYP-mediated metabolic pathways, substrates, substrate K_m_ values, positive control inhibitors and specific incubation conditions are shown in Additional file 1: Table S4. Multiple concentrations of each test compound (0.25 to 20 µM) or positive control inhibitor along with each substrate were added to a suspension of human liver microsomes in 0.1 M phosphate buffer (pH 7.4) containing 1 U/mL glucose-6-phosphate dehydrogenase at 37 °C. The final total organic solvent concentration (from the different spiking solutions) was 0.5% (v/v) for each sample. The reactions were initiated by the addition of an NADPH-regenerating system to give final concentrations of 1.4 mM NADP, 3.8 mM glucose-6-phosphate, and 3.5 mM MgCl_2_. Samples were quenched by the addition of ice-cold acetonitrile containing diazepam as the analytical internal standard. Concentrations of the substrate-specific metabolites in quenched samples were determined by LC–MS (Additional file 1: Table S5) relative to calibration standards prepared in quenched microsomal matrix. Control samples were included to confirm that the LC–MS assay of the specific metabolites was not affected by the presence of test compound (or potential test compound metabolites).

The inhibitory effect of each test compound and positive control inhibitor was based on the reduction in the formation of the specific CYP-mediated metabolite (represented as percent inhibition of enzyme activity) relative to metabolite formation in the absence of inhibitor (i.e. control for maximal enzyme activity). Where the inhibition of probe metabolite formation exceeded 50%, the inhibitor concentration resulting in 50% inhibition (IC_50_) was obtained by non-linear curve fitting of the percent inhibition vs inhibitor concentration using a 4-parameter sigmoidal function (GraphPad Prism, GraphPad Software, San Diego). Minimum and maximum inhibition values were constrained to 0 and 100%, respectively, unless reasonable model fitting could only be achieved without constraints. Where less than 50% inhibition was observed at the highest concentration tested (e.g. 20 µM in this assay), the IC_50_ value is reported as being > 20 µM. Where IC_50_ values could be measured, the inhibition constant (K_i_) was then calculated by dividing the IC_50_ value by (1 + [S]/K_m_) where [S] is the substrate concentration and K_m_ the Michaelis-Menten constant with an assumption of competitive inhibition. The K_m_ was determined under the same incubation conditions by measuring the rate of metabolite formation (pmol/min/mg protein) as a function of substrate concentration (Additional file 1: Table S4).

## Results

### Molecular properties

**Fig. 1:**
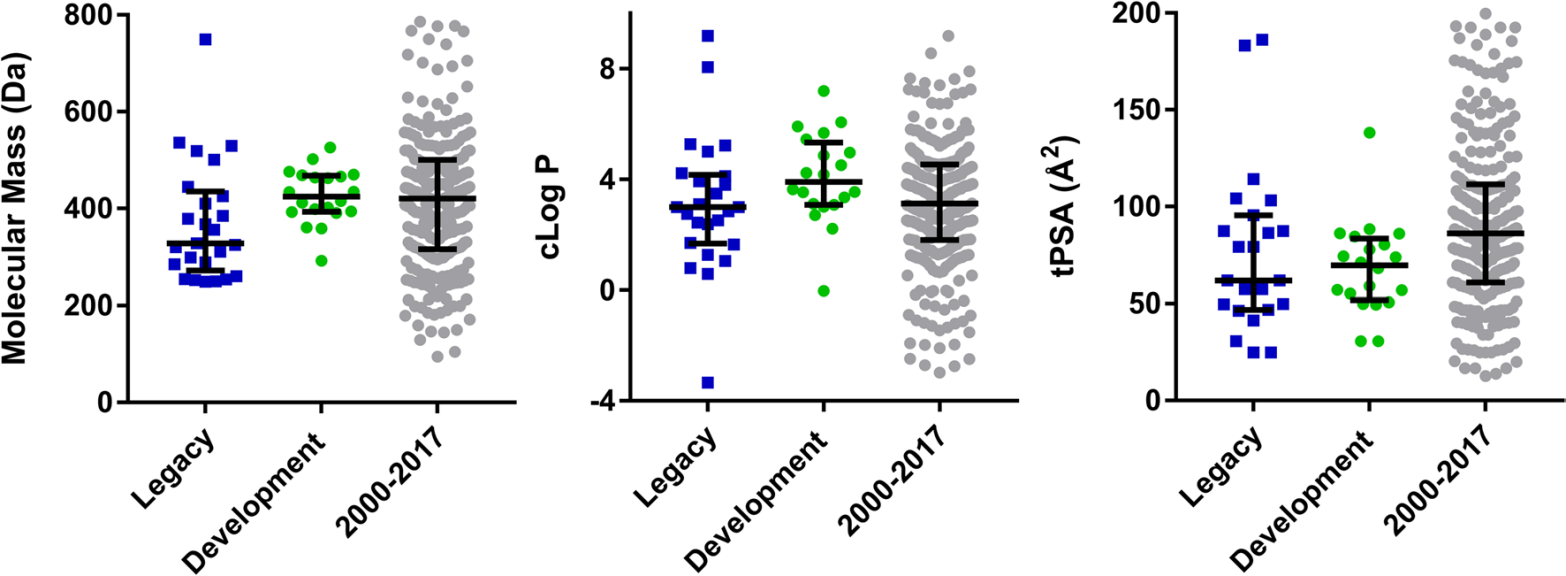
Molecular properties for the anti-malarial data sets and oral drugs launched between 2000 and 2017. Vertical bars represent the median and interquartile range

A comparison of the key properties for the legacy and development compounds is shown graphically in Fig. 1 and tabulated values are shown in Table 1. Median values for legacy and development compounds were not significantly different and median parameters were also comparable to those for oral drugs launched between 2000 and 2017.

**Table 1:** Calculated molecular properties (ChemAxon)

Compound	Mass (Da)	cLog P	HBD/HBA	tPSA (Å^2^)	FRB	AROM	Fsp^3^
Development compounds							
AQ-13	291.82	3.00	1/3	30.6	7	2	0.44
Artemisone	401.52	2.21	0/7	74.3	1	0	1.00
DSM265	415.33	5.68	1/4	55.1	4	3	0.21
DSM421	358.28	3.34	1/5	68.0	4	3	0.29
ELQ300	475.85	7.20	1/4	56.8	6	4	0.13
Ferroquine	433.76	3.54	1/3	30.6	7	2	0.26
JPC3210	398.45	4.51	2/3	49.7	6	2	0.48
KAF156	411.46	3.07	2/4	77.8	4	3	0.27
KAE609	390.24	4.17	3/2	56.9	0	3	0.21
M5717	462.57	3.53	1/5	58.9	7	3	0.41
MMV048	393.38	2.70	1/5	85.9	4	3	0.11
MMV052	525.73	5.91	2/6	73.8	4	1	0.81
MMV253	465.58	4.23	2/8	88.2	6	3	0.50
NPC1161B	434.36	4.86	2/4	71.0	8	3	0.32
OZ277	392.54	3.11	2/5	84.4	4	0	0.95
OZ439	469.62	5.44	0/6	49.4	5	1	0.79
P218	360.41	− 0.04	3/8	138	10	2	0.39
SJ733	468.41	3.64	1/4	86.1	5	3	0.17
Tafenoquine	463.50	4.97	2/5	80.3	10	3	0.38
TDD-E209	501.64	6.06	0/6	50.6	5	1	0.79
Legacy compounds							
Amodiaquine	355.87	3.80	2/4	49.6	6	3	0.25
Desethylamodiaquine	327.81	2.99	3/4	61.8	5	3	0.17
Artemether	298.38	3.48	0/5	46.2	1	0	1.00
Artesunate	384.43	3.10	1/7	103	5	0	0.89
Atovaquone	366.84	5.00	1/3	57.2	2	2	0.27
Azithromycin	749.00	2.44	5/13	183	7	0	0.97
Chloroquine	319.88	3.93	1/3	30.6	8	2	0.50
Chlorproguanil	288.18	2.99	5/5	87.3	2	1	0.27
Clindamycin	424.98	1.04	4/6	104	7	0	0.94
Dapsone	248.30	1.27	2/4	86.2	2	2	0.00
Dihydroartemisinin	284.35	2.84	1/5	57.2	0	0	1.00
Doxycyclin	444.44	− 3.34	6/9	186	2	1	0.41
Halofantrine	500.43	8.06	1/2	24.7	11	3	0.46
Lumefantrine	528.94	9.19	1/2	24.7	10	3	0.33
Mefloquine	378.32	4.11	2/3	49.7	4	2	0.47
Naphthoquine	409.96	5.22	3/4	61.8	5	3	0.38
Piperaquine	535.52	5.27	0/6	41.2	6	4	0.38
Primaquine	259.35	1.64	2/4	61.8	6	2	0.40
Proguanil	253.73	2.38	5/5	87.3	2	1	0.27
Cycloguanil	251.72	1.70	2/5	81.6	1	1	0.27
Pyrimethamine	248.71	2.75	2/4	79.1	2	2	0.17
Pyronaridine	518.06	4.22	2/7	79.0	7	4	0.38
Quinine	324.42	2.51	1/4	46.8	4	2	0.45
Sulfadoxine	310.33	0.58	2/7	114	4	2	0.17
Sulfamethoxazole	253.28	0.79	2/4	95.4	2	2	0.10

### Ionization and partitioning properties

**Fig. 2:**
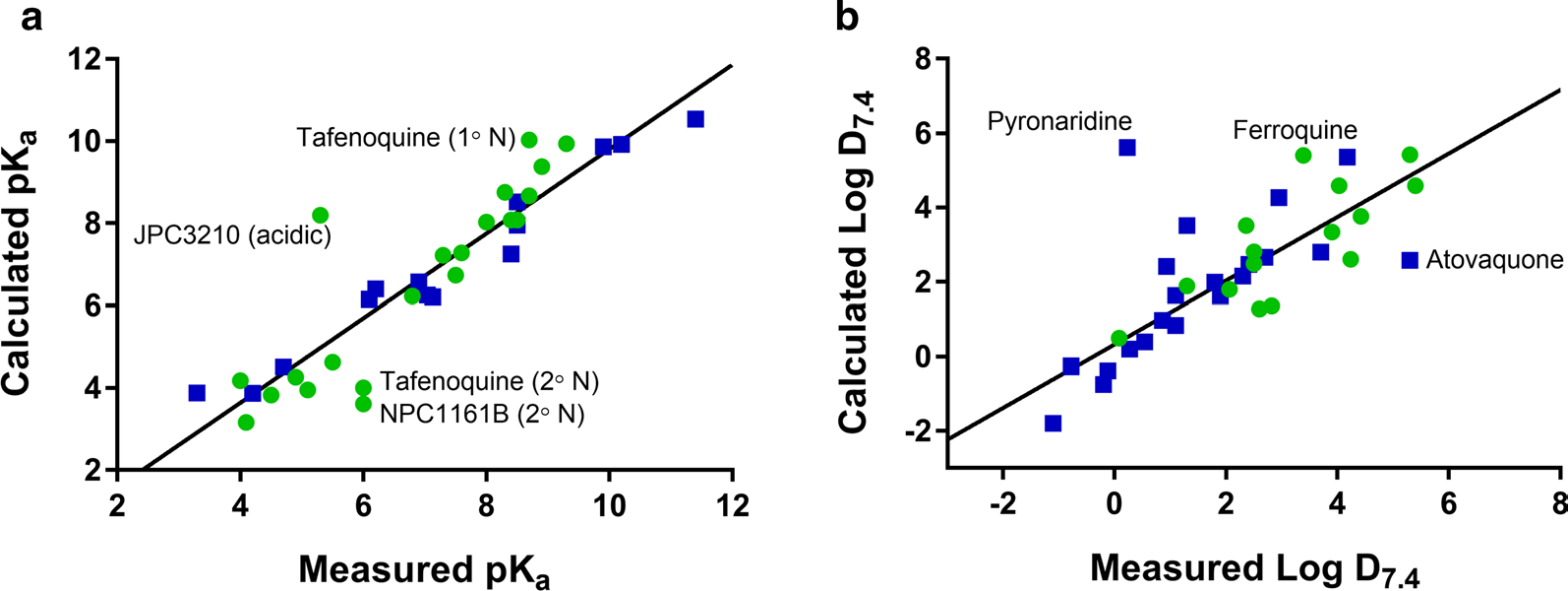
Relationship between calculated (using ADMET Predictor) and measured **a** pKa and **b** Log D_7.4_ values for development (green) and legacy (blue) compounds. Solid black lines represent the lines of best fit and labelled points are those that differed the most between the measured and calculated values

Calculated (using ADMET Predictor) and measured pKa values are shown in Table 2 and Fig. 2a. Calculated pKa values obtained using ChemAxon and ACD Labs (where available) are shown in Additional file 1: Table S6 for comparison. Of the 45 compounds in the data set, 12 are neutral at physiological pH whereas 26 are positively or partially positively charged weak bases, 4 are negatively charged weak acids, and 2 exist as zwitterions at physiological pH. Of the compounds that are neutral at physiological pH, 9 have weakly basic pKa values below 7.4 and are therefore positively charged at low pH conditions present in stomach. Several compounds (OZ439, TDD-E209, atovaquone, halofantrine, lumefantrine, naphthoquine) were sufficiently insoluble that pKa values could not be determined experimentally with the methods used in this work. Others (azithromycin, dapsone, doxycyclin, piperaquine, pyronaridine) contain multiple overlapping pKa values that precluded accurate measurement. For some compounds, there were two or more predicted pKa values within the range of 2–12 however only one ionization could be measured (KAE609, MMV253, M5717, JPC3210, amodiaquine, N-desethylamodiaquine, sulfadoxine).

**Table 2:** Calculated (ADMET predictor) and measured pKa and Log D_7.4_

Compound	Calculated pKa	Measured pKa^a^	Calculated Log D_7.4_	Measured Log D_7.4_^b^
Ionized or partially ionized bases at physiological pH				
Cycloguanil	10.5 (B)	11.4 ± 0.3 (B)	− 1.80	− 1.10
Doxycyclin	9.13 (B), 3.35 (A), 9.98 (A)	CND^e^	− 0.75	− 0.20
Pyronaridine	7.65 (B), 6.39 (B), 5.20 (B), 10.1 (A)	CND^e^	5.61	0.23
Proguanil	10.0 (B), 6.64 (B)	CND^c^	0.21	0.27
Primaquine	9.92 (B), 3.88 (B)	10.2 ± 0.12 (B), 3.3 ± 0.02 (B)	0.40	0.54
Chloroquine	9.86 (B), 7.25 (B)	9.9 ± 0.1 (B), 8.4 ± 0.1 (B)	2.42	0.93
Chlorproguanil	9.79 (B), 6.29 (B)	CND^c^	0.84	1.10
Azithromycin	8.72 (B), 7.63 (B)	CND^e^	1.64	1.10
AQ-13	9.63 (B), 7.28 (B)	7.6 ± 0.2 (B)	1.89	1.30
Desethylamodiaquine	10.3 (B), 6.21 (B), 8.14 (A)	8.5 ± 0.05 (B), 7.1 ± 0.01 (B)	3.51	1.30
Quinine	7.95 (B), 3.87 (B)	8.5 ± 0.05 (B), 4.2 ± 0.03 (B)	1.99	1.80
Clindamycin	7.44 (B)	7.1 ± 0.07 (B)	1.62	1.90
KAF156	8.08 (B), 3.83 (B)	8.4 ± 0.06 (B), 4.5 ± 0.02 (B)	1.80	2.06
M5717	8.67 (B), 6.23 (B), 2.54 (B), 10.9 (A)	8.7 ± 0.11 (B), 6.8 ± 0.04 (B)^g^	2.49	2.50 ^g^
OZ277	9.38 (B)	8.9 ± 0.16 (B)	1.28	2.60
Mefloquine	8.52 (B)	8.5 ± 0.04 (B)	2.66	2.70
Amodiaquine	7.95 (B), 6.25 (B), 10.3 (A)	7.0 ± 0.02 (B)	4.27	2.95
NPC1161B	9.94 (B), 3.61 (B)	9.3 ± 0.03 (B), 6.0 ± 0.01 (B)	3.24	CND^f^
Ferroquine	8.08 (B), 6.74 (B)	8.4 ± 0.06 (B), 7.5 ± 0.02 (B)	5.41	3.39
Naphthoquine	8.48 (B), 6.48 (B), 10.7 (A)	CND^d^	5.36	4.18
Tafenoquine	10.0 (B), 4.00 (B)	8.7 ± 0.09 (B), 6.0 ± 0.10 (B)	2.61	4.24
MMV253	8.03 (B), 4.63 (B), 3.03 (B), 2.52 (B)	8.0 ± 0.03 (B), 5.5 ± 0.03 (B)	3.76	4.42
MMV052	8.75 (B)	8.3 ± 0.06 (B)	4.58	5.40
Piperaquine	7.60 (B), 5.93 (B), 5.15 (B), 4.36 (B)	CND^e^	5.59	CND^f^
Halofantrine	9.20 (B)	CND^d^	5.78	CND^f^
TDD-E209	7.40 (B)	CND^d^	5.84	CND^f^
Lumefantrine	8.66 (B)	CND^d^	7.34	CND^f^
Ionized acids at physiological pH				
Sulfadoxine	2.01 (B), 6.40 (A)	6.2 ± 0.01 (A)	− 0.28	− 0.780
Sulfamethoxazole	6.15 (A)	6.1 ± 0.01 (A)	− 0.25	− 0.780
Artesunate	4.51 (A)	4.7 ± 0.04 (A)	− 0.38	− 0.120
Atovaquone	4.28 (A)	CND^d^	2.58	5.30
Zwitterionic or partially zwitterionic at physiological pH				
P218	7.22 (B), 4.26 (A)	7.3 ± 0.003 (B), 4.9 ± 0.002 (A)	0.49	0.080
JPC3210	8.20 (B), 10.5 (A)	5.3 ± 0.04 (A)	5.42	5.30
Neutral at physiological pH				
Dapsone	3.06 (B), 2.18 (B)	CND^e^	0.97	0.86
Dihydroartemisinin	NA	NA	2.16	2.30
DSM421	3.04 (B)	CND^c^	3.51	2.36 ^g^
Pyrimethamine	6.57 (B)	6.9 ± 0.10 (B)	2.47	2.41
MMV048	4.18 (B)	4.0 ± 0.07 (B)	2.81	2.50
Artemisone	5.22 (B)	CND^c^	1.36	2.82
Artemether	NA	NA	2.80	3.70
SJ733	3.16 (B), 10.7 (A)	4.1 ± 0.03 (B)	3.34	3.90
DSM265	3.23 (B)	CND^c^	4.59	4.03
KAE609	3.95 (B), 10.7 (A), 10.1 (A)	5.1 ± 0.02 (B)	4.36	CND^f^
OZ439	6.38 (B)	CND^d^	5.03	CND^f^
ELQ300	NA	NA	5.28	CND^f^

*A* acidic pKa, *B* basic pKa, *CND* could not determine, *NA* not applicable

^a^Values for pKa represent the mean ± SD for n = 3 titrations

^b^Values for Log D represent the average ratio for n = 2–3 replicate measurements of each partitioning phase (i.e. buffer or octanol); replicate measurements for each phase differed by less than 10%

^c^No ionization detected

^d^Solubility-limited

^e^Multiple overlapping pKa values

^f^Aqueous phase concentrations below the analytical LLQ

^g^Data for M5717 from [77] and for DSM421 from [78]

Overall, there was good agreement between the measured and calculated (ADMET Predictor) values for the majority of compounds, with the slope (1.03 ± 0.08) not differing significantly (p = 0.73) from unity (Fig. 2a). For a few compounds (artemisone, DSM265, DSM421), no ionizations could be detected in spite of the calculated pKa values being within a measurable range (i.e. 2–12) suggesting that ADMET Predictor overestimated the basicity of the nitrogens in these structures. This is supported by the solubility results for these three compounds (see below) which showed no major increase in solubility under low pH conditions (FaSSGF, pH 1.6) compared to more neutral pH (FaSSIF, pH 6.5). For these three compounds, the pKa calculations using ChemAxon (Additional file 1: Table S6) were more in line with the experimental results. Poor calculated predictions were obtained for JPC3210 (both acidic and basic groups), tafenoquine (less basic group), and NPC1161B (less basic group). For JPC3210 and tafenoquine, the ChemAxon (Additional file 1: Table S6) calculated values still differed considerably from the measured whereas the ChemAxon values for NPC1161B were somewhat more consistent with the measured values. As highlighted previously, it is unlikely that a single software package will be accurate for all compounds [44], however a rigorous assessment of the different calculation packages was outside the scope of this work.

Calculated (ADMET Predictor) and measured Log D_7.4_ values are shown in Table 2 and Fig. 2b. Calculated values using ChemAxon and ACD Labs (where available) are shown in Additional file 1: Table S6 for comparison. In general, the development compounds were somewhat more lipophilic than the legacy compounds, with 10 of 20 having calculated Log D_7.4_ values above 3.5 compared to only 6 out of 23 for the legacy compounds. Measured values were obtained for all compounds with the exception of halofantrine, lumefantrine, OZ439, ELQ300, KAE609 and piperaquine where concentrations in the aqueous phase were below the analytical limit of quantitation. Even though there was more scatter than for the pKa data, the slope (0.84 ± 0.092) of the calculated (ADMET Predictor) vs measured relationship (Fig. 2b) was not significantly different from unity (p = 0.074) suggesting that the calculated Log D_7.4_ values still provide a reasonable estimate of the true Log D_7.4_. Exceptions to this included pyronaridine, ferroquine and atovaquone and for each of these the ChemAxon or ACD calculated values (Additional file 1: Table S6) were somewhat more consistent with the measured values.

### Solubility

**Fig. 3:**
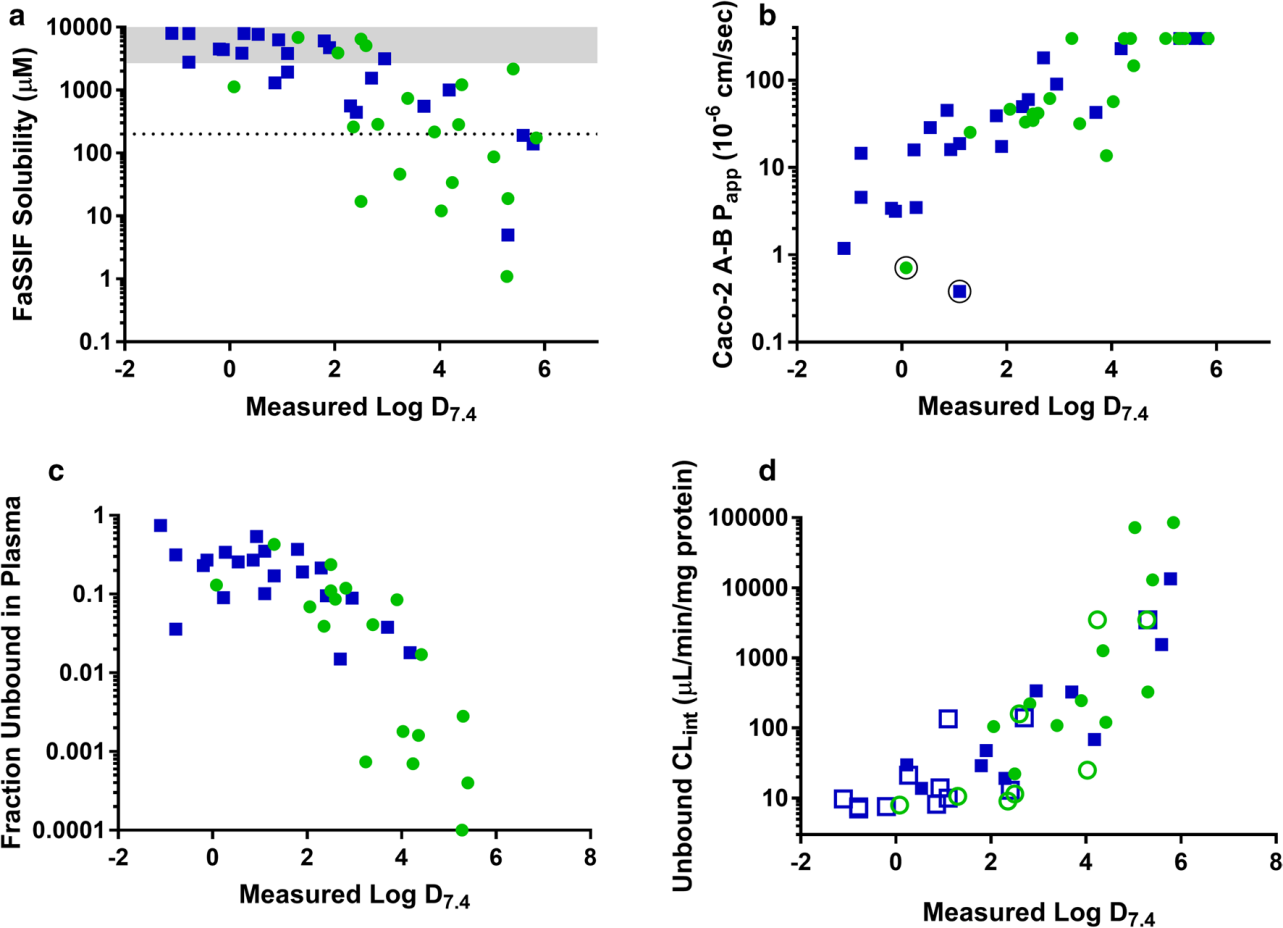
Relationship between **a** FaSSIF solubility (grey bar represents the maximum solubility range assessed), **b** Caco-2 permeability (circled symbols represent compounds with high efflux ratios), **c** fraction unbound in plasma, and **d** unbound intrinsic clearance (open symbols represent “less than” values) and measured Log D_7.4_. Where measured Log D values were not available, calculated (ADMET Predictor) values were used. Symbols represent data for development (green) and legacy (blue) anti-malarial compounds. Caco-2 P_app_ data for chloroquine, quinine, amodiaquine, naphthoquine, mefloquine, piperaquine, atovaquone and halofantrine are from Katneni et al. [31]

Measured solubility values determined in fasted state simulated gastric fluid (FaSSGF), fasted and fed state simulated intestinal fluids (FaSSIF and FeSSIF, both version 2 [30]) and phosphate buffered saline (pH 7.4) are shown in Table 3. Given the high prevalence of weak bases in the data set, it is not surprising that the majority of compounds had high solubility in simulated gastric fluid with most exceeding the maximum tested concentration of 2 mg/mL (2.6–8.1 mM). The notable exceptions to this were the neutral compounds or those showing minimal or no ionization (artemisone, DSM265, ELQ300, artemether), the weak acid (atovaquone) and the highly lipophilic weak bases (OZ439, MMV052, TDD-E209, halofantrine, lumefantrine). In general, solubility was considerably lower in FaSSIF and decreased with increasing Log D_7.4_ (Fig. 3a) but improved for most compounds in FeSSIF reflecting an increase in solubilization in the presence of bile salts and mixed micellar phases. Compounds that exhibited high solubility (> 2 mg/mL) in all media tested included the charged compounds (M5717, chloroquine, chlorproguanil, clindamycin, primaquine, and proguanil) and the very polar compound doxycycline. Development compounds KAF156 and P218 also had very good solubility (> 400–500 µg/mL) in all media tested.

**Table 3:** Measured solubility in fasted and fed state simulated intestinal fluids (FaSSIF and FeSSIF), fasted state simulated gastric fluid (FaSSGF), and pH 7.4 phosphate buffered saline

Compound	Solubility (µg/mL)/(µM)^a^			
	FaSSGF (1 h)	FaSSIF-V2 (5–6 h)	FeSSIF-V2 (5–6 h)	PBS_7.4_ (5–6 h)
Ionized or partially ionized bases at physiological pH				
Cycloguanil	> 2000/> 7950	> 2000/> 7950	533/2120	> 2000/> 7950
Doxycyclin	> 2000/> 4500	> 2000/> 4500	> 2000/> 4500	> 2000/> 4500
Pyronaridine	> 2000/> 3800	> 2000/> 3860	> 2000/> 3800	357/689
Proguanil	> 2000/> 7880	> 2000/> 7880	> 2000/> 7880	> 2000/> 7880
Primaquine	> 2000/> 7700	> 2000/> 7700	> 2000/> 7700	> 2000/> 7700
Chloroquine	> 2000/> 6250	> 2000/> 6250	> 2000/> 6250	> 2000/> 6250
Chlorproguanil	> 2000/> 6940	1100/3810	1670/5780	1420/4930
Azithromycin	1180/1570	1440/1930	> 2000/> 2670	> 2000/> 2670
AQ-13	> 2000/> 6850	> 2000/> 6850	> 2000/> 6850	1480/5070
Quinine	1925/5930	1960/6050	1930/5930	806/2480
Clindamycin	> 2000/> 4700	> 2000/> 4700	> 2000/> 4700	> 2000/> 4700
KAF156	> 2000/> 4850	1600/3880	> 2000/> 4850	560/1360
M5717	> 3000/> 6490^b^	> 3000/> 6490^b^	> 3000/> 6486^b^	> 3000/> 6490^b^
OZ277	> 2000/> 5000	> 2000/> 5100	> 2000/> 5000	198/504
Mefloquine	740/1960	584/1540	> 2000/> 5200	290/767
Amodiaquine	> 2000/> 5620	1120/3150	> 2000/> 5620	26.3/73.9
NPC1161B	1520/3500	19.9/45.8	> 2000/> 4600	5.44/12.5
Ferroquine	> 2000/> 4600	320/738	> 2000/> 4600	4.9/11
Naphthoquine	> 2000/> 4880	410/1000	941/2300	28.5/69.5
Tafenoquine	> 2000/> 2800	15.8/34.1	1310/2820	18.3/39.5
MMV253	> 2000/> 4300	569/1220	> 2000/> 4300	19.0/40.8
MMV052	13.0/24.7	1140/2170	> 2000/> 3800	49.6/94.3
Piperaquine	> 2000/> 3700	103/192	60.7/113	< 0.5/< 0.9
Halofantrine	12.7/25.4	69.9/140	739/1480	< 0.05/< 0.1
TDD-E209	211/421	87.2/174	> 2000/> 4000	0.056/0.112
Lumefantrine	12.6/23.8	0.063/0.119	14.4/27.2	< 0.05/< 0.1
Ionized acids at physiological pH				
Sulfadoxine	324/1040	858/2770	474/1530	> 1900/> 6400
Sulfamethoxazole	758/2990	> 2000/> 7900	> 2000/> 7900	> 2000/> 7900
Artesunate	321/835	1690/4390	1720/4490	1680/4370
Atovaquone	0.26/0.71	1.83/4.99	6.41/17.5	< 0.05/< 0.1
Zwitterionic or partially zwitterionic at physiological pH				
P218	> 2000/> 5550	408/1130	525/1460	1070/2960
JPC3210	213/535	7.60/19.1	131/329	0.800/2.01
Neutral at physiological pH				
Dapsone	946/3810	323/1300	423/1700	227/914
Dihydroartemisinin	140/492	159/559	246/865	130/457
DSM421	116/324^b^	92.7/259^b^	119/332^b^	85.5/239
Pyrimethamine	> 2000/> 8000	111/446	120/483	43/175
MMV048	1200/3040	6.73/17.1	7.47/19.0	4.52/11.5
Artemisone	89.1/222	115/286	278/692	86.1/214
Artemether	95/320	166/556	1040/3480	109/365
SJ733	> 2000/> 4270	101/216	119/254	115/246
DSM265	6.84/16.5^b^	5.12/12.3^b^	27.6/66.5^b^	2.04/4.9
KAE609	1540/3940	111/284	1240/3180	30.4/77.9
OZ439	79.0/168	40.6/87	526/1120	0.085/0.181
ELQ300	0.929/1.95	0.524/1.10	0.065/0.137	0.102/0.214

^a^Average of n = 2–3 technical replicates; replicate measurements differed by less than 10%

^b^Data for M5717 from [77], for DSM421 from [78], and for DSM265 from [79]

### Permeability

The measurement of flux across Caco-2 monolayers was used as a means to determine the apparent permeability coefficient (P_app_) which was then converted to a predicted effective human jejunal permeability (P_eff_) using a calibration plot of reported human P_eff_ values [34, 35] and measured Caco-2 P_app_ for a series of control compounds [31]. The general performance of the Caco-2 test system was assessed on the basis of the permeability data for the minimally permeable marker, lucifer yellow, the high permeability marker, propranolol, and the efflux ratio for a P-gp efflux marker, rhodamine 123 (Additional file 1: Table S7). The wide range of physicochemical properties across the data set necessitated the use of two different transport buffers consisting of either aqueous pH 7.4 buffer or human plasma as recently described [31]. Results for mass balance and P_app_ are shown in Table 4 and data for control compounds under the two conditions can be found in Katneni et al. [31].

**Table 4:** Caco-2 mass balance and bidirectional permeability coefficients

Compound	Matrix	A–B mass bal (%)	A–B P_app_^a^ (10^−6^ cm/s)	B–A mass bal (%)	B–A P_app_^a^ (10^−6^ cm/s)	Efflux ratio
Ionized or partially ionized bases at physiological pH						
Cycloguanil	Buffer	100	1.2 ± 0.070	100	0.77 ± 0.05	0.7
Doxycyclin	Buffer	99	3.4 ± 0.23	110	4.2 ± 0.29	1.2
Pyronaridine	Plasma	63	16 ± 2.2	87	41 ± 3.0	2.6
Proguanil	Buffer	82	3.5 ± 0.75	95	7.9 ± 0.51	2.3
Primaquine	Buffer	66	29 ± 4.5	99	34 ± 1.5	1.2
Chloroquine^b^	Plasma	61	16 ± 2.5	110	22 ± 4.6	1.4
Chlorproguanil	Plasma	79	19 ± 3.0	100	61 ± 7.4	3.2
Azithromycin	Buffer	98	0.37, 0.38	87	13 ± 3.9	33
AQ-13	Plasma	54	25 ± 2.3	92	32 ± 0.8	1.2
Quinine^b^	Buffer	70	39 ± 5.0	92	40 ± 4.5	1.0
Clindamycin	Buffer	75	17 ± 1.1	92	48 ± 9.1	2.8
KAF156	Plasma	86	47 ± 7.6	96	230 ± 17	4.9
M5717	Buffer	60	34 ± 2.6	81	39 ± 7.6	1.1
OZ277	Plasma	67	42 ± 2.8	100	70 ± 0.38	1.7
Mefloquine^b^	Plasma	70	180 ± 20	86	150 ± 10	0.83
Amodiaquine^b^	Plasma	81	90 ± 8.3	88	63 ± 9.6	0.7
NPC1161B	Plasma	76	> 300	97	> 300	–
Ferroquine	Plasma	66	32 ± 6.8	110	24 ± 4.1	0.8
Naphthoquine^b^	Plasma	72	230 ± 32	94	180 ± 10	0.78
Tafenoquine	Plasma	79	> 300	110	v300	–
MMV253	Plasma	84	150 ± 29	94	150 ± 23	1.0
MMV052	Plasma	77	> 300	110	> 300	–
Piperaquine^b^	Plasma	100	> 300	100	> 300	–
Halofantrine^b^	Plasma	83	> 300	87	> 300	–
TDD-E209	Plasma	93	> 300	100	> 300	–
Lumefantrine	Plasma	110	CND	110	CND	CND
Ionized acids at physiological pH						
Sulfadoxine	Buffer	93	15 ± 2.5	110	23 ± 2.8	1.6
Sulfamethoxazole	Buffer	96	4.5 ± 0.29	100	5.9 ± 0.26	1.3
Artesunate	Buffer	81	3.4, 2.9	84	2.8 ± 0.48	0.9
Atovaquone b	Plasma	99	> 300	97	> 300	–
Zwitterionic or partially zwitterionic at physiological pH						
P218	Buffer	96	0.71 ± 0.04	100	15 ± 1.7	21
JPC3210	Plasma	97	> 300	100	> 300	–
Neutral at physiological pH						
Dapsone	Buffer	86	45 ± 7.3	98	56 ± 1.3	1.2
Dihydroartemisinin	Buffer	85	50 ± 2.3	91	49 ± 1.2	1.0
DSM421^c^	Buffer	70	33 ± 2.5	98	46	1.4
Pyrimethamine	Buffer	75	60 ± 5.6	100	59 ± 2.9	1.0
MMV048	Buffer	82	41 ± 2.1	98	53 ± 1.9	1.3
Artemisone	Buffer	98	58.8, 64.5	99	47 ± 1.7	0.8
Artemether	Buffer	73	39.1, 46.3	95	42 ± 5.6	1.0
SJ733	Buffer	93	14.0, 13.4	99	50 ± 2.0	3.6
DSM265	Buffer	83	52.4, 69.7	93	49 ± 5.1	0.9
KAE609	Plasma	93	> 300	98	> 300	–
OZ439	Plasma	91	> 300	110	> 300	–
ELQ300	Plasma	99	> 300	110	> 300	–

*CND* could not determine

^a^Mean ± SD, n = 3–4 technical replicates

^b^Data from [31]

^c^Data from [78]

**Fig. 4:**
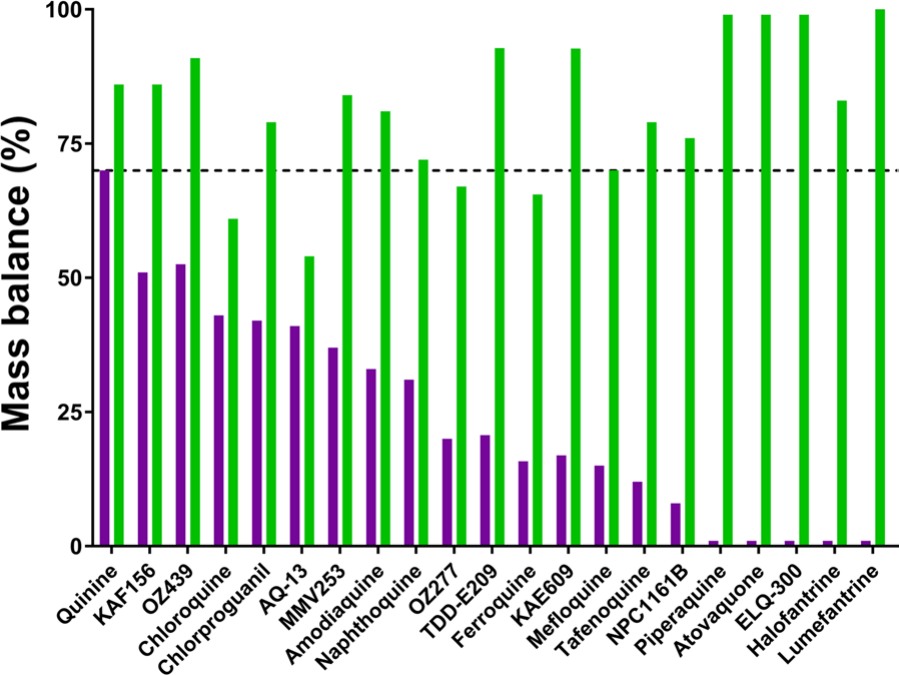
Mass balance for Caco-2 permeability experiments conducted using either pH 7.4 buffer (purple bars) or human plasma (green bars) as the transport matrix. Data for chloroquine, quinine, amodiaquine, naphthoquine, mefloquine, piperaquine, atovaquone and halofantrine are from Katneni et al. [31]

The use of plasma as the transport medium for the more lipophilic compounds significantly improved the mass balance as shown in Fig. 4 allowing permeability values to be measured even for the more lipophilic compounds. As expected, P_app_ increased with increasing Log D_7.4_ (Fig. 3b) with only a few of the more polar compounds showing low A-B P_app_ values (i.e. < 5 × 10^−6^ cm/s) including P218, azithromycin, cycloguanil, doxycycline, proguanil, and sulfamethoxazole. Several of the highly lipophilic compounds had P_app_ values in excess of 300 × 10^−6^ cm/s and, other than P218 and azithromycin (efflux ratios of 21 and 33, respectively), no compounds exhibited high efflux ratios (i.e. efflux ratios were generally less than 4).

**Fig. 5:**
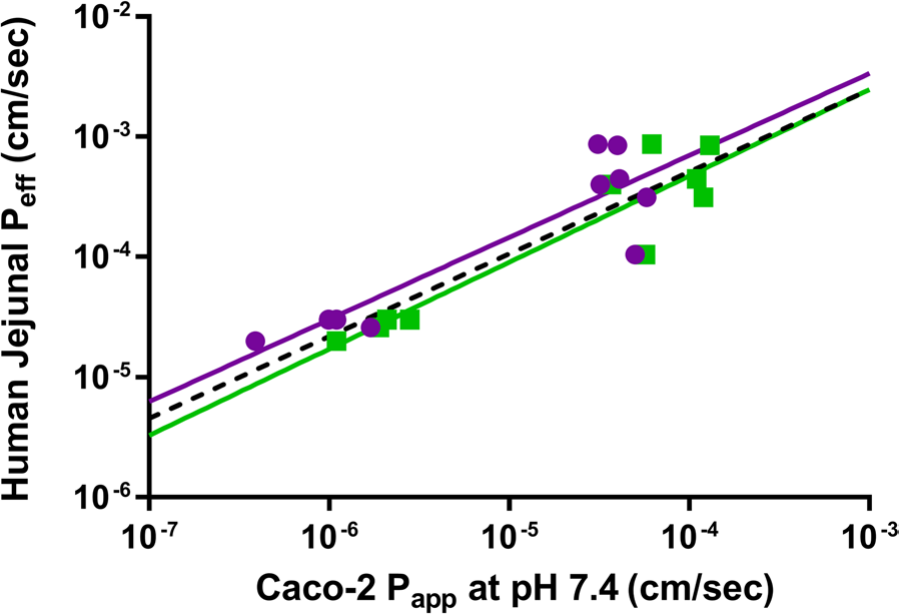
Relationship between human jejunal permeability (P_eff_ [34, 35]) and Caco-2 A-B apparent permeability (P_app_ [31]) at pH 7.4 measured using either pH 7.4 buffer (purple) or human plasma (green) as the transport medium. Solid lines represent the lines of best fit to the Log transformed data (for buffer: slope = 0.6989, y-int = − 0.3941; for plasma: slope = 0.7255, y-int = − 0.4239). The reported relationship from Sun et al. [45] is shown by the dashed black line (slope = 0.6836, y-int = − 0.5579)

Figure 5 illustrates the relationship between human jejunal P_eff_ values from the literature [34, 35] and the measured Caco-2 A-B P_app_ for control compounds using either pH 7.4 buffer or plasma as the transport medium [31]. Also shown is the relationship from Sun et al. [45] for passively permeating compounds using pH 7.4 buffer as the Caco-2 transport medium showing the similarity in the relationships across the different studies. As shown previously, P_app_ values are typically higher using plasma as the transport medium compared to buffer due to improved sink conditions [31] and as a result the relationship using plasma is shifted marginally to the right.

### Solubility limited absorbable dose

Measured solubility (FaSSIF) and predicted human permeability (P_eff_ based on Caco-2 P_app_ values and the calibration data shown in Fig. 5) were used to estimate the solubility limited absorbable dose (SLAD, Table 5) as described previously [32]. SLAD values ranged from less than 10 mg for the compounds showing very low FaSSIF solubility (in spite of good permeability for several of these) to greater than 2 g for compounds demonstrating both high solubility and high permeability.

**Table 5:** Estimated solubility limited absorbable dose (SLAD)

Compound	Permeability			Solubility/dose	
	Predicted human P_eff_ (10^−4^ cm/s)	Permeability classification^a^	An	S_si_ (µg/mL)	SLAD (mg)
Ionized or partially ionized bases at physiological pH					
Doxycyclin	0.70	Intermediate	1.0	> 2000	> 1000
Pyronaridine	1.4	High	1.6	> 2000	> 1610
Proguanil	0.71	Intermediate	1.0	> 2000	> 1000
Primaquine	3.0	High	3.6	> 2000	> 3560
Chloroquine	1.4	High	1.6	> 2000	> 1620
Chlorproguanil	1.5	High	1.8	1100	998
Azithromycin	0.16	Low	1.0	1440	720
AQ-13	1.9	High	2.2	> 2000	> 2250
Quinine	3.7	High	4.4	1960	4280
Clindamycin	2.1	High	2.5	> 2000	> 2530
KAF156	2.9	High	3.5	1600	2780
M5717	3.4	High	4.0	> 3000	> 6050
OZ277	2.7	High	3.2	> 2000	> 3240
Mefloquine	7.7	High	9.2	584	2690
Amodiaquine	4.7	High	5.6	1120	3130
Ferroquine	2.2	High	2.7	320	426
Naphthoquine	9.2	High	11	410	2250
Tafenoquine	11	High	13	15.8	105
MMV253	6.8	High	8.1	569	2300
MMV052	11	High	13	1140	7580
Piperaquine	11	High	13	103	685
Halofantrine	11	High	13	69.9	466
TDD-E209	11	High	13	87.0	579
Lumefantrine	CND	CND	CND	CND	CND
Ionized acids at physiological pH					
Sulfadoxine	1.9	High	2.2	858	959
Sulfamethoxazole	0.85	Intermediate	1.0	> 2000	> 1000
Artesunate	0.66	Intermediate	1.0	1690	845
Atovaquone	11	High	13	1.83	12
Zwitterionic or partially zwitterionic at physiological pH					
P218	0.24	Low	1.0	408	204
JPC3210	11	High	13	7.60	51
Neutral at physiological pH					
Dapsone	4.1	High	4.9	323	786
Dihydroartemisinin	4.4	High	5.2	159	413
DSM421	3.3	High	3.9	93.0	182
Pyrimethamine	4.9	High	5.9	111	326
MMV048	3.8	High	4.5	6.70	15
Artemisone	5.0	High	6.0	115	345
Artemether	3.9	High	4.7	166	388
SJ733	1.8	High	2.2	101	109
DSM265	4.8	High	5.7	5.10	14
KAE609	11	High	13	111	738
OZ439	11	High	13	40.6	270
ELQ300	11	High	13	0.520	3

*CND* could not determine

^a^Classifications based on [80] using control compounds which have > 85% absorption (high), 50–84% absorption (intermediate) or < 50% absorption (low) and previously published relationships [31]

### Binding and whole blood partitioning

Binding properties were assessed in human plasma, Albumax medium (used for the majority of in vitro *P. falciparum* activity assays), 10% FCS in DMEM (used in several standard parasite in vitro assays), and human liver microsomes (Table 6). For Albumax, DMEM/FCS and HLMs, an ultracentrifugation method was used to separate bound and free fractions.

**Table 6:** Binding to plasma and media proteins

Compound	Fraction unbound ± SD			
	Human Plasma^a^	Albumax^a^	10% FCS in DMEM^a^	HLM^a^
Ionized or partially ionized bases at physiological pH				
Cycloguanil	0.75 ± 0.040	0.88 ± 0.057	0.94 ± 0.064	0.72 ± 0.083
Doxycyclin	0.23 ± 0.090^b^	0.41 ± 0.023	0.44 ± 0.070	0.93 ± 0.045
Pyronaridine	0.090 ± 0.012^b^	0.69 ± 0.023	Not assessed	0.29 ± 0.018
Proguanil	0.34 ± 0.010	0.80 ± 0.021	Not assessed	0.33 ± 0.014
Primaquine	0.26 ± 0.023	0.96 ± 0.056	0.78 ± 0.025	0.56 ± 0.050
Chloroquine	0.54 ± 0.048	0.97 ± 0.069	Not assessed	0.50 ± 0.032
Chlorproguanil	0.10 ± 0.0070	0.50 ± 0.020	Not assessed	0.052 ± 0.004
Azithromycin	0.34, 0.35^b^	0.76 ± 0.065	Not assessed	0.70 ± 0.060
AQ-13	0.43 ± 0.022	0.85 ± 0.028	Not assessed	0.66 ± 0.048
Desethylamodiaquine	0.17 ± 0.0020	0.75 ± 0.074	Not assessed	not assessed
Quinine	0.37 ± 0.040^b^	0.69 ± 0.090	Not assessed	0.57 ± 0.036
Clindamycin	0.19 ± 0.011	0.65 ± 0.066	Not assessed	0.67 ± 0.084
KAF156	0.069 ± 0.013^b^	0.38 ± 0.017	0.477 ± 0.032	0.14 ± 0.0060
M5717	0.24 ± 0.0020^b^	0.55 ± 0.041	0.638 ± 0.065	0.50 ± 0.041
OZ277	0.086 ± 0.0080	0.29 ± 0.021	Not assessed	0.044 ± 0.0050
Mefloquine	0.015 ± 0.0010^b^	0.27 ± 0.019	Not assessed	0.050 ± 0.0060
Amodiaquine	0.089 ± 0.0080	0.67 ± 0.033	Not assessed	0.91 ± 0.094
NPC1161B	0.00074 ± 0.00016^c^	0.035 ± 0.0020	0.043 ± 0.004	CND^e^
Ferroquine	0.041 ± 0.0010^c^	0.52 ± 0.031	0.364 ± 0.026	0.27 ± 0.032
Naphthoquine	0.018 ± 0.0010^c^	0.27 ± 0.035	Not assessed	0.21 ± 0.015
Tafenoquine	0.00070 ± 0.00017^c^	CND^e^	0.038 ± 0.001	0.0020 ± 0.00030
MMV253	0.017 ± 0.00020^c^	0.48 ± 0.026	Not assessed	0.11 ± 0.017
MMV052	0.00040 ± 0.00010^c^	0.014 ± 0.0010	Not assessed	0.0010 ± 0.00010
Piperaquine	0.0003 ± 0.0001^c^	0.019 ± 0.0040	0.092 ± 0.0030	0.013 ± 0.0020
Halofantrine	< 0.0001^c^	0.0080 ± 0.0010	Not assessed	0.0020 ± 0.00020
TDD-E209	< 0.0001^c^	0.0070 ± 0.0010	Not assessed	0.00030 ± 0.000010
Lumefantrine	< 0.0001^c^	CND^e^	Not assessed	CND^e^
Ionized acids at physiological pH				
Sulfadoxine	0.036 ± 0.0010	0.50 ± 0.030	0.86 ± 0.044	0.93 ± 0.086
Sulfamethoxazole	0.32 ± 0.024	0.67 ± 0.120	0.87 ± 0.058	1.00 ± 0.033
Artesunate	0.27 ± 0.0080^d^	0.14 ± 0.0070^d^	0.72 ± 0.026	CND^d^
Atovaquone	< 0.0001^c^	CND^e^	CND^e^	~ 0.0020
Zwitterionic or partially zwitterionic at physiological pH				
P218	0.13 ± 0.013	0.41 ± 0.022	0.684 ± 0.017	0.88 ± 0.041
JPC3210	0.0028 ± 0.00020^c^	0.21 ± 0.012	0.227 ± 0.012	0.029 ± 0.0030
Neutral at physiological pH				
Dapsone	0.27 ± 0.019	0.63 ± 0.11	Not assessed	0.86 ± 0.059
Dihydroartemisinin	0.21 ± 0.010^d^	0.34 ± 0.018^d^	Not assessed	0.87 ± 0.10^d^
DSM421	0.039 ± 0.007	0.43 ± 0.020	0.615 ± 0.043	0.77 ± 0.065^f^
Pyrimethamine	0.095 ± 0.0010^b^	0.46 ± 0.090	0.48 ± 0.034	0.54 ± 0.097
MMV048	0.11 ± 0.0070^b^	0.46 ± 0.022	0.653 ± 0.060	0.61 ± 0.014
Artemisone	0.12 ± 0.0060	0.46 ± 0.013	Not assessed	0.44 ± 0.024
Artemether	0.038 ± 0.0090^c^	0.46 ± 0.039	Not assessed	0.50 ± 0.052
SJ733	0.085 ± 0.012^c^	0.40 ± 0.040	Not assessed	0.58 ± 0.069
DSM265	0.0018 ± 0.00010^c^	0.18 ± 0.0050^f^	0.366 ± 0.041	0.28 ± 0.0070^f^
KAE609	0.0016 ± 0.00010^c^	0.044 ± 0.0040	Not assessed	0.019 ± 0.0010
OZ439	< 0.0001^c^	0.010 ± 0.0010	Not assessed	0.0010 ± 0.00010
ELQ300	0.00010 ± 0.000010^c^	CND^e^	0.001 ± 0.001	0.002 ± 0.00010

*CND* could not determine

^a^Mean ± SD, n = 3–4 replicates; ultracentrifugation used unless indicated otherwise

^b^RED device with diluted plasma correcting for the dilution factor with 6 h dialysis

^c^RED device with diluted plasma correcting for the dilution factor; presaturation of device and 24 h dialysis

^d^Instability evident; where values are given they represent an estimate only

^e^Compound not detected in free fraction

^f^Data for DSM265 from [79] and for DSM421 from [78]

Since the physicochemical and protein binding properties varied considerably across the data set, it was necessary to use a range of methods to minimize experimental artefacts for plasma (e.g. compound adsorption to the dialysis chamber or membrane, or slow equilibration) and obtain measurable unbound concentrations. Preliminary plasma f_u_ values for control and anti-malarial compounds using the different methods are shown in Additional file 1: Tables S8 and S9, respectively, and the final f_u_ values for the anti-malarial compounds obtained under the optimized conditions are shown in Table 6. Fraction unbound values ranged from < 0.0001 for the most lipophilic compounds to > 0.4 for some of the more polar compounds and generally correlated with Log D_7.4_ as shown in Fig. 3c.

Table 7 shows values for the whole blood to plasma partitioning ratio across the data set. While many of the values are close to 1, some of the compounds showing very high plasma protein binding (e.g. DSM265, ELQ-300, JPC3210, atovaquone, lumefantrine) have restricted distribution into red blood cells (B/P value 0.5–0.6) while others (4-aminoquinolines, proguanil and chlorproguanil) appear to concentrate in red blood cells (B/P > 3).

**Table 7:** Whole blood to plasma partitioning

Compound	B/P^a^	Haematocrit (Gender)
Ionized or partially ionized bases at physiological pH		
Cycloguanil	0.71 ± 0.07	0.48 (M)
Doxycyclin	0.78 ± 0.06	0.43 (M)
Pyronaridine	9.0 ± 0.83	0.48 (M)
Proguanil	3.30 ± 0.14	0.46 (M)
Primaquine	0.82 ± 0.07	0.44 (M)
Chloroquine	3.5 ± 0.09	0.42 (F)
Chlorproguanil	3.3 ± 0.38	0.43 (M)
Azithromycin	1.6 ± 0.19	0.48 (M)
AQ-13	3.15 ± 0.20	0.42 (M)
Desethylamodiaquine	3.26 ± 0.35	0.50 (M)
Quinine	0.67 ± 0.02	0.44 (M)
Clindamycin	0.61 ± 0.04	0.42 (M)
KAF156	1.3 ± 0.02	0.42 (F)
M5717	1.3 ± 0.02	0.42 (F)
OZ277	1.10 ± 0.10	0.46 (M)
Mefloquine	1.1 ± 0.07	~ 0.43 (M)
Amodiaquine	1.1 ± 0.18	~ 0.43 (M)
NPC1161B	1.09 ± 0.07	0.44 (M)
Ferroquine	1.6 ± 0.15	~ 0.43 (M)
Naphthoquine	1.14 ± 0.10	~ 0.43 (M)
Tafenoquine	1.3 ± 0.04	~ 0.43 (M)
MMV253	1.0 ± 0.05	~ 0.43 (M)
MMV052	1.6 ± 0.14	~ 0.43 (M)
Piperaquine	0.57 ± 0.05	~ 0.43 (M)
Halofantrine	0.68 ± 0.06	0.43 (M)
TDD-E209	0.51 ± 0.08	0.44 (M)
Lumefantrine	0.48 ± 0.09	0.48 (M)
Ionized acids at physiological pH		
Sulfadoxine	0.57 ± 0.03	0.46 (M)
Sulfamethoxazole	0.65 ± 0.07	0.43 (M)
Artesunate	CND^b^	0.44 (M)
Atovaquone	0.52 ± 0.03	0.44 (M)
Zwitterionic or partially zwitterionic at physiological pH		
P218	0.56 ± 0.03	~ 0.43 (M)
JPC3210	0.54 ± 0.04	~ 0.43 (M)
Neutral at physiological pH		
Dapsone	1.1 ± 0.06	0.43 (M)
Dihydroartemisinin	CND^b^	0.42 (M)
DSM421	0.53 ± 0.03	0.44 (M)
Pyrimethamine	0.84 ± 0.03	0.43 (M)
MMV048	0.77 ± 0.03	0.42 (F)
Artemisone	CND^b^	0.44 (M)
Artemether	CND^b^	0.42 (M)
SJ733	0.72 ± 0.02	0.42 (F)
DSM265	0.54 ± 0.02	0.44 (M)
KAE609	0.66 ± 0.04	0.42 (F)
OZ439	0.78 ± 0.05	0.44 (M)
ELQ300	0.53 ± 0.05	0.44 (M)

^a^Mean ± SD, n = 3–4 measurements

^b^CND = could not determine, unstable in blood and plasma

### In vitro metabolism

Intrinsic clearance (CL_int_) was assessed in human liver microsomes and values for total and unbound CL_int_ are shown in Table 8. Out of 45 compounds in the dataset, 22 showed less than 15% degradation over the 60 min incubation precluding the determination of CL_int_. Of the compounds where degradation was detected, intrinsic clearance was relatively low (< 20 µL/min/mg) for most compounds, however unbound CL_int_ values varied considerably reflecting the high degree of binding for many compounds in the data set. Fraction unbound values could not be measured for NPC1161B, artesunate, or lumefantrine precluding estimation of unbound CL_int_ values. Data for control compounds included in each assay are shown in Additional file 1: Table S10. Figure 3d illustrates that unbound in vitro CL_int_ values were highly correlated with Log D_7.4_.

**Table 8:** *In vitro* metabolism in human liver microsomes

Compound	HLM CL_int_ (µL/min/mg)^a^	Unbound CL_int_ (µL/min/mg)
Ionized or partially ionized bases at physiological pH		
Cycloguanil	< 7^b^	< 9.72^c^
Doxycyclin	< 7^b^	< 7.53^c^
Pyronaridine	8.6	29.7
Proguanil	< 7^b^	< 21.2^c^
Primaquine	7.70	13.8
Chloroquine	< 7^b^	< 14^c^
Chlorproguanil	< 7^b^	< 135^c^
Azithromycin	< 7^b^	< 10^c^
AQ-13	< 7^b^	< 10.6^c^
Desethylamodiaquine	13.3	CND^d^
Quinine	16.5	28.9
Clindamycin	32.0	47.8
KAF156	14.7	105
M5717	11.1	22.2
OZ277	< 7^b^	< 159
Mefloquine	< 7^b^	< 140^c^
Amodiaquine	308	339
NPC1161B	< 7^b^	CND
Ferroquine	29.2	108
Naphthoquine	14.4	68.6
Tafenoquine	< 7^b^	< 3500^c^
MMV253	13.2	120
MMV052	13.0	13,000
Piperaquine	20.1	1540
Halofantrine	26.9	13,500
TDD-E209	25.5^e^	85,000
Lumefantrine	< 7^b^	CND^d^
Ionized acids at physiological pH		
Sulfadoxine	< 7^b^	< 7.53
Sulfamethoxazole	< 7^b^	< 7.00
Artesunate	146	CND^d^
Atovaquone	< 7^b^	< 3500^c^
Zwitterionic or partially zwitterionic at physiological pH		
P218	< 7^b^	< 7.95^c^
JPC3210	9.50	328
Neutral at physiological pH		
Dapsone	< 7^b^	< 8.14^c^
Dihydroartemisinin	16.6	19.1
DSM421	< 7^b^	< 9.09^c^
Pyrimethamine	< 7^b^	< 13^c^
MMV048	< 7^b^	< 11.5^c^
Artemisone	97.7	222
Artemether	163	326
SJ733	142	245
DSM265	< 7^b,e^	< 25^c^
KAE609	24.1	1270
OZ439	72.1	72,100
ELQ300	< 7^b^	< 3500^c^

^a^Average of n = 2 replicates

^b^< 15% loss over 60 min

^c“^less than” CL_int_ values corrected for measured microsomal binding

^d^CND = could not determine, plasma or microsomal f_u_ value not available

^e^Data for DSM265 from [79] and for TDD-E209 from [81]

### CYP inhibition

The ability of compounds to inhibit the five major CYP isoforms was assessed in human liver microsomes. Data for the anti-malarial compounds are presented in Table 9 and positive control inhibitors are shown in Additional file 1: Table S11. The majority of compounds showed no inhibition up to the highest concentration tested (20 µM). The most frequently inhibited isoform was CYP2D6 where 17 compounds had IC_50_ values below 10 µM with 10 below 3 µM. Surprisingly, only 5 compounds showed evidence of inhibiting CYP3A4 with IC_50_ values in the range of 3–13 µM.

**Table 9:** CYP inhibition in human liver microsomes

Compound	IC_50_ (µM) (% inhibition at max conc)/K_i_ (µM)					
	CYP1A2	CYP2C9	CYP2C19	CYP2D6	CYP3A4 (test)	CYP3A4 (midaz)
Ionized or partially ionized bases at physiological pH						
Cycloguanil	> 20 (nmi)	> 20 (nmi)	> 20 (nmi)	6.0/3.7	> 20 (nmi)	> 20 (nmi)
Doxycyclin	CND^a^	> 20 (nmi)	> 20 (nmi)	> 20 (nmi)	> 20 (22%)	> 20 (nmi)
Pyronaridine	> 20 (30%)	> 20 (nmi)	> 20 (nmi)	1.2/0.75	> 20 (nmi)	> 20 (28%)
Proguanil	> 20 (nmi)	> 20 (nmi)	> 20 (27%)	3.5/2.2	> 20 (24%)	> 20 (nmi)
Primaquine	< 0.25/< 0.15	> 20 (20%)	> 20 (34%)	> 20 (33%)	> 20 (nmi)	> 20 (31%)
Chloroquine	> 20 (nmi)	> 20 (nmi)	> 20 (nmi)	6.1/3.8	> 20 (nmi)	> 20 (24%)
Chlorproguanil	> 20 (nmi)	> 20 (nmi)	> 20 (39%)	1.4/0.87	> 20 (41%)	> 20 (nmi)
Azithromycin	> 20 (nmi)	> 20 (nmi)	> 20 (29%)	> 20 (nmi)	> 20 (nmi)	> 20 (nmi)
AQ-13	> 20 (nmi)	> 20 (nmi)	> 20 (nmi)	13/8.3	> 20 (nmi)	> 20 (nmi)
Desethylamodiaquine	> 18 (nmi)	> 18 (nmi)	> 18 (nmi)	2.6/1.6	CND^a^	> 18 (nmi)
Quinine	> 20 (nmi)	> 20 (nmi)	> 20 (nmi)	6.0/3.7	> 20 (nmi)	> 20 (nmi)
Clindamycin	> 20 (nmi)	> 20 (nmi)	> 20 (32%)	> 20 (nmi)	> 20 (nmi)	> 20 (nmi)
KAF156	> 20 (nmi)	19/14	> 20 (40%)	1.2/0.75	2.7/1.6	2.5/1.8
M5717	> 20 (nmi)	> 20 (nmi)	> 20 (nmi)	> 20 (nmi)	> 20 (nmi)	> 20 (nmi)
OZ277	> 20 (nmi)	> 20 (nmi)	> 20 (nmi)	> 20 (nmi)	> 20 (46%)	> 20 (26%)
Mefloquine	> 20 (nmi)	> 20 (nmi)	> 20 (nmi)	16/10	> 20 (29%)	> 20 (nmi)
Amodiaquine	> 20 (27%)	> 20 (nmi)	> 20 (nmi)	0.88/0.55	> 20 (nmi)	> 20 (nmi)
NPC1161B	6.5/3.8	> 20 (37%)	9.6/5.7	> 20 (39%)	13.6/8.2	> 20 (37%)
Ferroquine	> 20 (nmi)	> 20 (nmi)	> 20 (nmi)	0.83/0.52	> 20 (42%)	7.3/5.2
Naphthoquine	> 20 (33%)	> 20 (nmi)	> 20 (nmi)	1.1/0.68	> 20 (38%)	> 20 (41%)
Tafenoquine	> 20 (20%)	14/10	> 20 (42%)	> 20 (35%)	3.8/2.3	> 20 (40%)
MMV253	> 20 (nmi)	> 20 (nmi)	CND^a^	> 20 (35%)	> 20 (37%)	CND^a^
MMV052	> 20 (nmi)	> 20 (nmi)	> 20 (nmi)	> 20 (nmi)	> 20 (nmi)	> 20 (nmi)
Piperaquine	> 20 (nmi)	CND^a^	> 20 (nmi)	> 20 (nmi)	> 20 (nmi)	4.5/3.2
Halofantrine	> 20 (nmi)	> 20 (nmi)	> 20 (nmi)	0.27/0.19	> 20 (19%)	> 20 (24%)
TDD-E209^b^	> 20 (nmi)	> 20 (nmi)	> 20 (nmi)	> 20 (nmi)	> 20 (43%)	> 20 (33%)
Lumefantrine	> 20 (nmi)	> 20 (nmi)	> 20 (nmi)	2.9/1.8	> 20 (nmi)	> 20 (nmi)
Ionized acids at physiological pH						
Sulfadoxine	> 20 (nmi)	> 20 (nmi)	> 20 (nmi)	> 20 (nmi)	> 20 (nmi)	> 20 (nmi)
Sulfamethoxazole	CND^a^	> 20 (nmi)	> 20 (nmi)	> 20 (nmi)	> 20 (nmi)	> 20 (nmi)
Artesunate	20/12	> 20 (nmi)	> 20 (nmi)	> 20 (nmi)	20 (nmi)	> 20 (nmi)
Atovaquone	> 20 (nmi)	> 20 (26%)	> 20 (nmi)	> 20 (nmi)	> 20 (nmi)	> 20 (nmi)
Zwitterionic or partially zwitterionic at physiological pH						
P218	> 20 (nmi)	> 20 (nmi)	> 20 (nmi)	> 20 (nmi)	> 20 (nmi)	> 20 (nmi)
JPC3210	> 20 (nmi)	> 20 (nmi)	> 20 (nmi)	0.70/0.43	> 20 (nmi)	> 20 (nmi)
Neutral at physiological pH						
Dapsone	> 20 (nmi)	> 20 (nmi)	> 20 (nmi)	> 20 (nmi)	> 20 (nmi)	> 20 (nmi)
Dihydroartemisinin	11/6.2	> 20 (nmi)	> 20 (nmi)	> 20 (nmi)	> 20 (nmi)	> 20 (nmi)
DSM421^b^	> 20 (nmi)	> 20 (16%)	> 20 (nmi)	> 20 (33%)	> 20 (nmi)	CND^a^
Pyrimethamine	> 20 (nmi)	> 20 (nmi)	> 20 (nmi)	> 20 (36%)	> 20 (nmi)	> 20 (nmi)
MMV048	> 20 (nmi)	> 20 (nmi)	> 20 (nmi)	> 20 (16%)	> 20 (nmi)	CND^a^
Artemisone	NA	NA	NA	NA	NA	NA
Artemether	> 20 (nmi)	> 20 (nmi)	> 20 (nmi)	> 20 (nmi)	> 20 (37%)	> 20 (39%)
SJ733	> 20 (nmi)	> 20 (33%)	> 20 (38%)	16/9.6	> 20 (37%)	> 20 (33%)
DSM265^b^	> 20 (nmi)	> 20 (25%)	> 20 (19%)	7.1/4.4	> 20 (34%)	CND
KAE609	4.5/2.7	5.5/4.0	< 0.25/< 0.15	5.9/3.7	> 20 (30%)	> 20 (nmi)
OZ439	> 20 (nmi)	> 20 (nmi)	> 20 (nmi)	> 20 (nmi)	5.1/3.1	12/8.6
ELQ300	> 20 (nmi)	7.5/5.4	> 20 (41%)	8.0/5.0	> 20 (nmi)	CND^a^

*nmi* no measurable inhibition

^a^CND = could not determine; inhibition profiles not well defined

^b^Data for DSM265 from [79], for DSM421 from [78] and for TDD-E209 from [81]

## Discussion

The objective of this work was to collect in vitro ADME data using standardized conditions for a set of legacy and development anti-malarial compounds to facilitate discovery and development activities, and more specifically, to enhance modelling and simulation approaches being applied to predictions of human dose, pharmacokinetic profiles and drug–drug interactions. The parameters evaluated included those that represent input parameters for PBPK modelling, namely pKa, Log D_7.4_, solubility in biorelevant media, effective human intestinal permeability, plasma protein binding, blood to plasma partitioning, unbound intrinsic clearance and inhibition of the major CYP isoforms. In addition, binding values were obtained with media used for in vitro activity assessment for the major parasite assay formats such that intrinsic unbound activity can be compared across platforms and incorporated into pharmacokinetic/pharmacodynamic (PKPD) modelling. Included in the data set were 23 legacy drugs (including three that have been withdrawn due to toxicity issues), two active metabolites (desethylamodiaquine and cycloguanil), and 20 compounds in preclinical (a few which have since been discontinued) or clinical development (including recently introduced compounds, tafenoquine and OZ277).

### Methodology considerations

Data for measured pKa and Log D_7.4_ suggested that the calculated values generated using the ADMET Predictor software provided a reasonable estimation for most compounds, however there were still cases where the calculated values differed significantly from the measured values. Given the importance of these two parameters as key determinants of tissue-to-plasma partitioning ratios in PBPK modelling, the results suggest that measured values for pKa and Log D_7.4_ should be generated and used whenever possible.

The two most challenging properties to measure were permeability and plasma protein binding due to the broad range of physicochemical properties across the data set and the fact that each of these assays is prone to artefacts for highly lipophilic compounds. For Caco-2 cell permeability, non-specific adsorption to the transport chambers and high retention in the cell monolayer due to the absence of effective sink conditions can result in very poor mass balance and an underestimation of the resulting permeability coefficient [31]. As shown in Fig. 4, many of the compounds in the data set had mass balance values well below 50% when a simple pH 7.4 aqueous buffer was used as the transport medium precluding the measurement of reliable P_app_ values. However, if plasma was used as the transport medium (with subsequent correction of P_app_ for the fraction unbound) [31], mass balance was improved to more than 70–80% for most compounds giving P_app_ values that are more in line with the expected permeation properties based on their molecular and physicochemical properties (as reviewed by [46]).

Given that commonly used in vitro permeability assays were not available at the time that most of the legacy anti-malarials were developed, there are few reports of measured apparent permeability values for these compounds in the literature. Even where values have been reported previously (Additional file 1: Table S12), interlaboratory variation in test conditions and measured P_app_ values makes it difficult to directly compare results [47]. Notwithstanding these issues, three compounds that have moderate to good solubility and good mass balance in the current studies (e.g. dihydroartemisinin, artemisone, and artesunate, Additional file 1: Table S12) showed similar P_app_ values compared to those reported previously. For several other compounds that exhibited poor mass balance using a standard aqueous buffer as the transport medium (e.g. naphthoquine, piperaquine, mefloquine, pyronaridine and amodiaquine, Fig. 4), measured P_app_ values using plasma as the transport buffer were considerably higher than those reported previously (Additional file 1: Table S12). For example, low to moderate P_app_ values have been reported for piperaquine, mefloquine, and amodiaquine [48, 49] whereas each of these was found to be highly permeable under the revised conditions.

To assess plasma protein binding, three different approaches were used depending on the matrix (e.g. media or plasma). Initially, ultracentrifugation was used for media and plasma based on a method adapted from that previously published by Nakai et al. [36]. Compared to equilibrium dialysis, this method has the advantage of not being plagued by non-specific compound adsorption to a dialysis membrane and is relatively straight forward and quick to conduct. For all media except plasma, the ultracentrifugation method was considered suitable given that the method was shown to remove > 99.9% of the total protein (assessed using the Bradford assay as described previously [50, 51]) and these media do not contain lipoproteins which have variable sedimentation rates [52]. The control of pH for the bicarbonate-buffered media (e.g. plasma, Albumax and DMEM/FCS) was necessary but could be readily achieved by equilibration of the samples and rotor in a suitable CO_2_ atmosphere (either 5 or 10% depending on the media) prior to sealing the rotor. For plasma, the ultracentrifugation method may potentially underestimate f_u_ for very highly bound compounds or those that associate with lipoproteins due to residual protein in the supernatant fraction following ultracentrifugation [36]. Under the current conditions, supernatant protein concentrations following ultracentrifugation of neat plasma represented only about 0.2% of the total plasma protein concentration (assessed using the Bradford assay as described previously [50, 51]). However, total triglyceride levels (assessed using a colorimetric triglyceride assay kit, GPO-PAP, Roche Diagnostics) in the supernatant were approximately 17% of those in total plasma suggesting that the method does not satisfactorily remove the total lipoprotein pool. This is not likely an issue for many compounds but could be significant for highly lipophilic compounds that associate with the lipoprotein fraction, such as halofantrine [53]. Given these potential limitations, a conservative approach was applied and the UC binding results accepted only if the measured plasma f_u_ values were equal to or greater than 0.1.

For compounds that were more highly bound in plasma (nominally those with f_u_ < 0.1), a RED method was incorporated based on previous publications [39, 40, 54]. To increase the likelihood of being able to measure unbound concentrations in the dialysate, 10% human plasma diluted with pH 7.4 phosphate buffer was used with subsequent correction of the measured f_u_ for the dilution factor [41]. It should be noted that the use of diluted plasma can lead to errors for compounds where the binding is very low as there will be minimal difference between the measured post-dialysis unbound and total concentrations. The use of diluted plasma is also prone to error if a compound is exclusively bound to α-1 acid glycoprotein due to the potential for saturation under the dilute conditions, although as highlighted previously, this situation is not common [40].

For the very highly bound compounds (nominally f_u_ < 0.01), additional measures were taken to reduce the impact of non-specific adsorption to the dialysis membrane and accelerate the attainment of steady state equilibrium. These included (i) the use of a 24-h presaturation period exposing the dialysis unit and membrane to compound concentrations exceeding the expected unbound concentration, (ii) inclusion of a low concentration of compound (similar to the expected unbound concentration) in the dialysate at the start of the dialysis period, and (iii) a 24 h dialysis period [39, 40]. These conditions were considered the most stringent that could be practically incorporated under routine experimental conditions.

In several cases (e.g. for the control compounds propranolol, ketoprofen and warfarin and the anti-malarials ferroquine, KAF156, MMV048, DSM421, SJ733, amodiaquine, OZ277, pyrimethamine, sulfadoxine), values obtained using the RED method were comparable to those measured by UC even though the f_u_ value was less than the conservative cut-off of 0.1 (Additional file 1: Tables S8 and S9). As shown in Additional file 1: Table S9, compounds showing low binding (i.e. f_u_ > 0.1) generally had measured f_u_ values that were in very good agreement with previously reported values (e.g. AQ-13, desethylamodiaquine, chloroquine, clindamycin, dapsone, doxycycline, M5717, primaquine, proguanil, quinine, and sulfamethoxazole). It should be noted that different batches of pooled plasma will introduce a degree of variability in the data even if the results for two methods are comparable.

For several of the more highly bound lipophilic compounds (e.g. DSM265, KAE609, tafenoquine, JPC3210, NPC1161B, MMV052, artemether, piperaquine), considerably lower f_u_ values were obtained using the RED method (in either the 6 or 24 h dialysis format) compared to the UC method (Additional file 1: Table S9). Where literature reported values were obtained using equilibrium dialysis or erythrocyte partitioning methods, the current values using the RED method (6 or 24 h dialysis) were generally consistent with reported results (e.g. amodiaquine, artemether, mefloquine, pyrimethamine, sulfadoxine, Additional file 1: Table S9). In some cases (e.g. DSM265, KAE609, naphthoquine), the extra precautions taken to presaturate the dialysis unit and accelerate the attainment of steady state appeared to be unnecessary as the RED f_u_ values were comparable for the 6 h and 24 h dialysis conditions. However, in other cases (e.g. tafenoquine, ELQ300, NPC1161B, MMV052, piperaquine) the additional measures allowed the measurement of unbound concentrations where they could not be measured without these more extreme conditions. Equally, for several of the highly bound compounds, the RED method incorporating presaturation and a long dialysis period gave considerably lower f_u_ values than those previously reported using other methods (e.g. JPC3210, tafenoquine, lumefantrine, piperaquine, Additional file 1: Table S9).

As pointed out previously [39], it would be preferable to use multiple conditions to confirm convergence of the f_u_ to a common value to provide confidence in the measured result. Ideally, one would also measure fraction unbound using multiple pooled plasma aliquots, however these additional precautions were not practical for the number of compounds examined here. Even with the more conservative presaturation RED method, f_u_ values were still unable to be measured for halofantrine, lumefantrine, OZ439 and TDD-E209, and atovaquone. This could be due to extremely high binding, residual effects of non-specific adsorption, lack of steady state equilibrium under these experimental conditions, or a combination of these factors.

In this work, the binding measurements in microsome and Albumax media were conducted using the ultracentrifugation method, however the RED assay is equally applicable for these media. Issues related to non-specific adsorption and slow equilibration with more lipophilic compounds still need to be considered for these matrices in the same way as for plasma as described above. Out of the 45 compounds in the dataset, roughly half exhibited minimal degradation in hepatic microsomes under the standard conditions used here. Incubations were not extended past the 60 min time point given the risk of decreasing enzyme activity with time [55]. For a subset of compounds, the microsomal protein concentration was increased (to 2 mg/mL) in an attempt to obtain measurable levels of degradation (i.e. > 15%), but this approach was not successful. It is unknown at this stage whether the apparent stability results from inherently low unbound intrinsic clearance, or high microsomal binding, or a combination of the two, however it is noted that of the 22 compounds that showed minimal degradation, 8 were also highly bound to microsomal proteins (and of these 8, 5 had measured or calculated LogD_7.4_ values > 3), likely giving a false indication of their metabolic stability. These results emphasize the need for improved methods to assess intrinsic clearance for compounds that are highly metabolically stable and/or highly bound to microsomal proteins. Although not assessed as part of this work, additional studies should also be conducted using S9 fraction and hepatocytes to rule out the potential for non-CYP-mediated metabolic liabilities (e.g. due to metabolism by aldehyde or xanthine oxidases, or conjugative biotransformation).

### Physicochemical property trends

Consistent with numerous reports in the literature regarding the links between lipophilicity and ADME properties [56–61], there was a notable correlation between several of the measured properties and Log D_7.4_. As shown in Fig. 3, both solubility and fraction unbound decreased, and permeability and unbound intrinsic clearance increased, with increasing Log D_7.4_ above a value of about 2. Except for one compound (P218), each of the development compounds had high permeability consistent with their relatively high Log D_7.4_, and accordingly, many had quite poor solubility in FaSSIF (≤ 100 µg/mL). The solubility-limited absorbable dose (SLAD) was calculated as described previously [32] taking into account both the predicted effective human permeability and the solubility properties. For the development compounds, 11 out of 20 had SLAD values below 400 mg (Table 5).

While the efficacious clinical dose for most of these compounds has not yet been finalized, the SLAD estimations highlight the likelihood that formulation approaches may be necessary to overcome solubility-limited absorption should these compounds continue to progress. However, as highlighted previously [32, 62], the solubility estimates based only on FaSSIF are likely conservative given that most of these compounds are weak bases and therefore, their intestinal solubility will also be impacted by their solubility in gastric fluids which is considerably higher than that in FaSSIF in the majority of cases (Table 3). Most of these compounds also exhibited greatly improved solubility in FeSSIF compared to FaSSIF likely as a result of solubilization by colloidal species present in the medium. This raises the potential for a food effect if the dose is high and if enabling formulations are not used to mitigate the solubility limitations.

Given that the clinical dose for the development compounds is either unknown or not yet fixed, and the solubility properties over the full pH range of 1–7.5 have not been determined, these compounds cannot strictly be classified according to either the Biopharmaceutics Classification System (BCS [33]) or the Biopharmaceutics Drug Disposition Classification System (BDDCS [63]). However, given the available data it is likely that, with the exception of P218, all of the development compounds will fall into either Class I or II based on either the BCS (i.e. high permeability) or the BDDCS (i.e. metabolism as the predominant clearance pathway). In contrast, several of the legacy drugs (e.g. sulfamethoxazole, doxycyclin, azithromycin, proguanil, and cycloguanil) have high polarity (PSA > 75 Å^2^), low Log D_7.4_ (Log D < 0), low permeability and high solubility placing them into BCS/BDDCS Class III. This is consistent with several of these compounds being subject to predominantly renal and/or biliary clearance mechanisms.

### Case studies relating physicochemical properties to current target product profiles

Current target product profiles for new anti-malarials to treat uncomplicated malaria aim to improve patient compliance through shorter treatment regimens (< 3 days and ideally with a single administration) and maximize efficacy and reduce transmission by maintaining effective concentrations for a period sufficient to achieve a 6–12 Log reduction in parasite burden [64]. This goal places a high burden on the pharmacokinetic properties to deliver the required half-life, and in many cases, this comes at the expense of good physicochemical properties. A high dose may also be required to extend the duration of pharmacological exposure (depending on potency) which further exacerbates issues related to less than ideal physiochemical properties.

One example of the impact of physicochemical properties on duration of exposure is the recently FDA approved 8-aminoquinoline, tafenoquine, designed at the Walter Reed Army Institute of Research (WRAIR) to increase the half-life of the structural analogue, primaquine ([65] and references therein). Primaquine (PQ) and tafenoquine (TQ) are the only available drugs that are effective in treating both pre-erythrocytic and erythrocytic forms of *Plasmodium vivax*, including the relapsing hypnozoite form. Compared to PQ which requires daily administration for 14 days, TQ achieves similar efficacy with only a single dose, representing a significant improvement with respect to dosing convenience. Physicochemical and pharmacokinetic properties of TQ and PQ are summarized in Table 10. The considerably longer half-life of TQ (~ 15 days) compared to PQ (~ 7 h) results from its higher lipophilicity, higher plasma protein binding, higher apparent oral volume of distribution, and lower apparent oral clearance (resulting from reduced free concentrations due to high binding). Not surprisingly, the solubility of TQ free base in FaSSIF is considerably lower than that for PQ, but TQ solubility increases considerably in FeSSIF, consistent with the known increase in exposure of TQ when administered with food. The absorption of TQ is not compromised by the lower solubility due to its formulation as the succinate salt to improve solubility/dissolution properties and recommendation that it is administered with food [66].

**Table 10:** Physicochemical and pharmacokinetic properties for selected anti-malarials

Property	8-Aminoquinolines		Peroxides		
	Primaquine^a^	Tafenoquine^a^	DHA^b^	OZ277^b^	OZ439^b^
Log D_7.4_	0.54	4.24	2.3	2.6	> 5
Plasma f_u_	0.26	0.0007	0.21	0.086	< 0.0001
FaSSGF solubility (µg/mL)	> 2000	> 2000	140	> 2000	79
FaSSIF solubility (µg/mL)	> 2000	15.8	159	> 2000	40.6
FeSSIF solubility (µg/mL)	> 2000	1310	246	> 2000	526
Half-life (h)	7	360	1	3	> 40
V/F (L)	277	1600	385	929	1570
CL/F (L/h)	28	3.0	272	184	41
Food effect	no	yes	no	no	yes

^a^Pharmacokinetic data for TQ from [82] and PQ from [83]

^b^Pharmacokinetic data for DHA from [84], OZ277 from [85], and OZ439 from [70]

A second example of the link between half-life and physicochemical properties is the synthetic ozonide, OZ439 [67]. Similar to the artemisinin derivatives and the first generation ozonide, OZ277 ([68]), OZ439 contains a relatively unique peroxide pharmacophore which is responsible for its potent and fast acting activity on all erythrocytic stages of *P. falciparum* and *P. vivax*. Physicochemical and pharmacokinetic properties of DHA, OZ277, and OZ439 are summarized in Table 10. Both dihydroartemisinin (DHA) and OZ277 suffer from a short in vivo half-life of approximately 1 h or 3 h, respectively, necessitating a 3-day treatment regimen for each (in combination with a longer acting partner drug). For both compounds, this short half-life is due in part to rapid breakdown of the peroxide moiety in blood as described previously [67, 69]. As a result of the substantially higher lipophilicity, higher plasma protein binding, higher apparent oral volume of distribution, and lower apparent oral clearance (resulting from a combination of higher plasma protein binding and reduced blood-mediated degradation [67]), OZ439 has a considerably longer half-life (> 40 h) than either DHA or OZ277. The improved half-life of OZ439 comes at the expense of solubility, resulting in an increase in exposure when administered with food [70] and significant formulation challenges [71]. This is further confounded by the need for a relatively high dose (> 500 mg) to achieve parasite clearance with a single administration.

## Conclusions

The methods used in these studies have been designed to provide the necessary in vitro data to support PBPK modelling activities for new anti-malarials and address practical issues common to several of the assays used for this purpose. The work highlights the challenges that are often encountered with compounds that cover a wide range of physicochemical characteristics and emphasizes that for many of these platforms, it is unlikely that a single method format will be universally applicable to all compounds. Two other useful platforms that have not been included in this work are the assessment of CYP and UGT reaction phenotyping, and time-dependent CYP inhibition as both of these are important for predicting potential drug–drug interactions. Methods and conditions for these studies are well described in the literature (see reviews [72] for reaction phenotyping and [73] for time-dependent inhibition). Further work is needed to develop suitable and practical methods that can be used to estimate human intrinsic clearance and metabolic pathways for highly bound, highly stable compounds since the standard methodology is often unsuitable for this purpose.

In recent years there has been an increased focus on the discovery of anti-malarial compounds and combination treatments that can be given as a single oral dose to improve patient compliance and reduce treatment costs compared to the current 3-day dosing regimens for most anti-malarials [4, 64]. While the benefits of this goal are clear, there is an associated requirement for an extended duration of pharmacological exposure to achieve a 6–12 Log reduction in parasitaemia [64]. Furthermore, both components of a combination treatment need to have matched durations of coverage to avoid exposing parasites to suboptimal concentrations of a single agent which would facilitate the development of resistance [74]. This requirement means that compounds need to have very low clearance (as a result of low unbound intrinsic clearance rather than high protein binding) and a moderate to high volume of distribution (typically driven by increasing lipophilicity, the introduction of one or more basic centres, or a combination of the two [75]) to achieve a long half-life. Depending on the potency, a relatively high dose may also be necessary to maintain exposure for the required duration. Such a high total exposure also increases the need for a wide safety margin. Given these challenges, predictive modelling tools are likely to play an increasing role in identifying risks and developing early mitigation strategies in late stage discovery and translational development of new anti-malarial drugs [16].

As illustrated by the current data set and the two examples given, there has been a trend toward the discovery of more lipophilic compounds to drive long half-life. In contrast to several of the legacy compounds which low Log D_7.4_ and low to moderate permeability, only one of the development compounds was poorly permeable suggesting that neither passive permeability or transporters are likely to limit oral absorption or hepatic elimination of the development compounds [63, 76]. It is unsurprising that these physicochemical trends come at the expense of good aqueous solubility in several cases. This emphasizes that there is significant scope, and substantial need, for the development of alternative formulation and delivery approaches to address solubility-limited absorption which are cost effective, stable under the harsh environmental conditions of climatic zone 4, and which can be used across all patient populations, including children and infants. The emphasis of current discovery projects is to achieve an extended duration of exposure by maximizing potency (to maintain a low effective dose) and minimizing unbound intrinsic clearance (to extend the half-life) without compromising physicochemical properties such as solubility, and several compounds currently in clinical development fulfil these objectives.

## Supplementary information

**Additional file 1.** Additional tables.

## Data Availability

All data and additional information are provided in the manuscript or additional information.

## Abbreviations

ACT: artemisinin-based combination therapy
PBPK: physiologically-based pharmacokinetic
ICH: International Conference on Harmonization
VIS: volunteer infection study
pKa: ionization constant
Log D_7.4_: Logarithm of the octanol/pH 7.4 buffer partition coefficient
FaSSIF: fasted-state simulated intestinal fluid
FeSSIF: fed-state simulated intestinal fluid
FaSSGF: fasted-state simulated gastric fluid
PBS: phosphate buffered saline
SD: standard deviation
S.E.: standard error
P_app_: apparent permeability coefficient
P_eff_: effective human jejunal permeability coefficient
V: fluid volume of small intestine
S_SI_: solubility in small intestine
M_p_: permeability multiplier
An: absorption number
t_res_: residence time in small intestine
R: radius of small intestine
SLAD: solubility limited absorbable dose
DMEM: Dulbecco’s modified Eagle’s medium
UC: ultracentrifugation
RED: rapid equilibrium dialysis
f_u_: fraction unbound
C_total_: total concentration in plasma or medium
C_unbound_: unbound concentration in plasma or medium
k: apparent first order rate constant for substrate depletion
HLM: human liver microsome
CL_int_: intrinsic clearance
CYP: cytochrome P450
IC_50_: concentration resulting in 50% inhibition of CYP activity
K_m_: Michaelis–Menten constant
K_i_: enzyme inhibition constant
