# Supplementary material for: An in vitro toolbox to accelerate anti-malarial drug discovery and development
Source: Malar J. 2020 Jan 2;19:1. doi: 10.1186/s12936-019-3075-5 (PMC6941357; doi:10.1186/s12936-019-3075-5)
Supplement: Supplementary file 1 — Additional file 1. Additional tables. [file 12936_2019_3075_MOESM1_ESM.pdf]

An *in vitro* toolbox to accelerate antimalarial drug discovery and development

Susan A. Charman*, Alice Andreu, Helena Barker, Scott Blundell, Anna Campbell, Michael Campbell, Gong Chen, Francis C.K. Chiu, Elly Crighton, Kasiram Katneni, Julia Morizzi, Rahul Patil, Thao Pham, Eileen Ryan, Jessica Saunders, David M. Shackleford, Karen L. White, Lisa Almond, Maurice Dickins, Dennis A. Smith, Joerg J. Moehrle, Jeremy N. Burrows, Nada Abla

ADDITIONAL INFORMATION

Table S1 Structures and salt forms

Compound	Salt	Biological Target	Structure	Status
Amodiaquine	Dihydrochloride	haemozoin formation	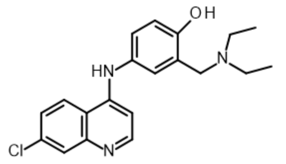	Approved
AQ-13	Dihydrochloride	assumed to be inhibition of haemozoin formation	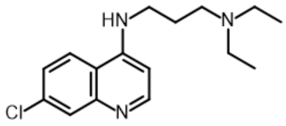	Phase II
Artemether	--	not known (likely poly-pharmacology)	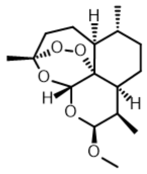	Approved
Artemisone	--	not known (likely poly-pharmacology)	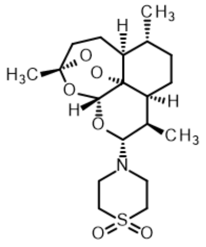	Phase II
Artesunate	Sodium	prodrug	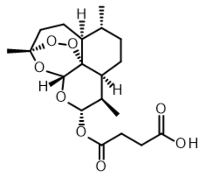	Approved
Atovaquone	--	Pfcyt bc_1 Qo	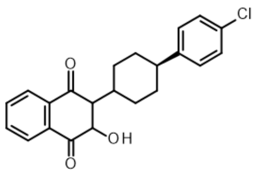	Approved
Azithromycin	--	70S ribosome	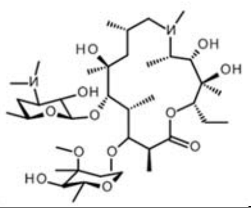	Approved

Chloroquine	Diphosphate	haemozoin formation	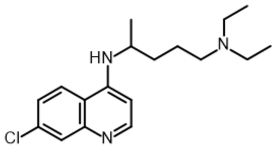	Approved
Chlorproguanil	Hydrochloride	Unknown (metabolite is Chlorcyclo-guanil - PfDHFR inhibitor)	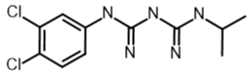	Approved (withdrawn as part of the combination Lapdap)
Clindamycin	Hydrochloride	70S ribosome	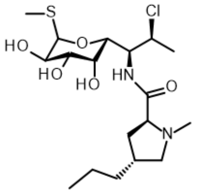	Approved
Cycloguanil	Nitrate	PfDHFR	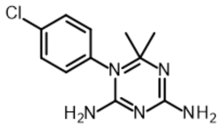	Active metabolite of proguanil
Dapsone	--	PfDHPS	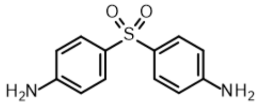	Approved (withdrawn as part of the combination Lapdap-dapsone showed unacceptable safety profile)
Dihydro-artemisinin	--	not known (likely poly-pharmacology)	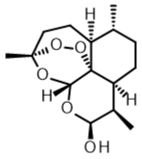	Approved
Doxycyclin	Hyclate	ribosome	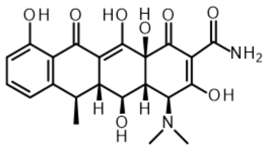	Approved
DSM265	--	PfDHODH	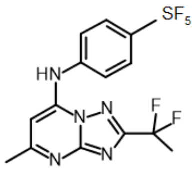	Phase II

DSM421	--	PfDHODH	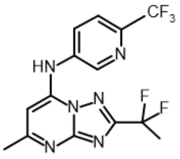	Program stopped due to toxicity
ELQ300	--	Pfcyt bc_1 Qi	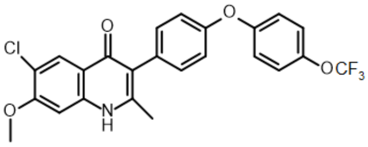	Preclinical
Ferroquine	--	haemozoin formation	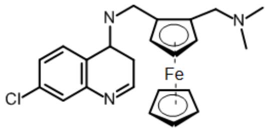	Phase II
Halofantrine	Hydrochloride	haemozoin formation	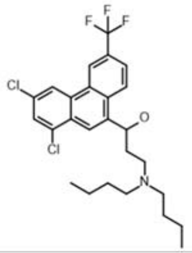	Withdrawn due to safety concerns
JPC3210	--	not known	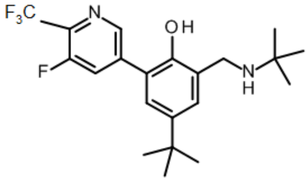	Preclinical
KAE609	--	PfATP4	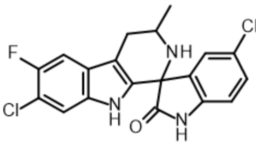	Phase II
KAF156	Hydrochloride	PfCARL (resistance marker)	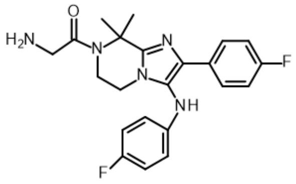	Phase II

Lumefantrine	--	haemozoin formation	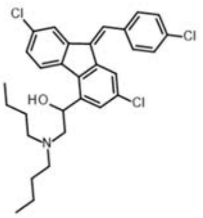	Approved
M5717	Base or Fumarate	PfeEF2	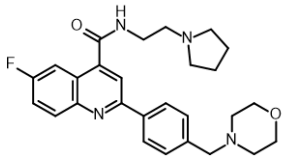	Phase I
Mefloquine	Hydrochloride	haemozoin formation	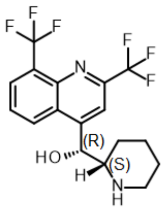	Approved
MMV048	--	PfPI(4)K	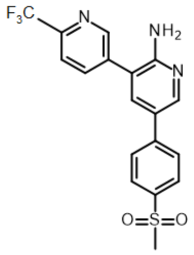	Phase II
MMV052	Tosylate or Phosphate	not known (likely poly-pharmacology)	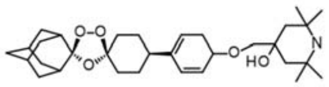	Program discontinued
MMV253	--	V-type ATPase (resistance marker)	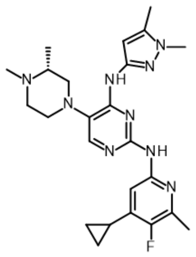	Phase I

Naphthoquine	Diphosphate	haemozoin formation	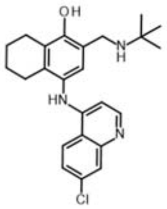	No stringent regulatory approval but part of marketed combination ARCO
N-Desethyl-amodiaquine	--	haemozoin formation	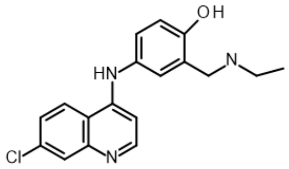	active metabolite of amodiaquine
NPC1161B	--	not known	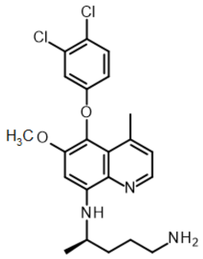	Preclinical
OZ277	Maleate	not known (likely poly-pharmacology)	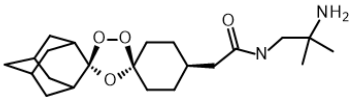	Approved (India)
OZ439	Mesylate	not known (likely poly-pharmacology)	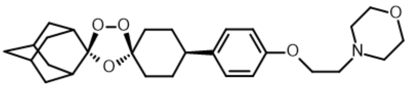	Phase II
P218	Hydrochloride	PfDHFR	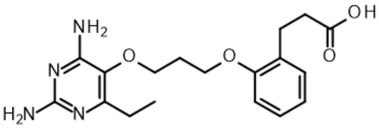	Phase I
Piperaquine	Tetraphosphate	haemozoin formation	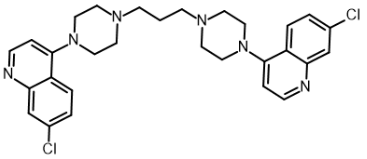	Approved

Primaquine	Diphosphate	not known	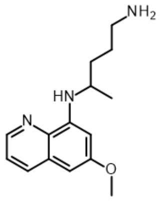	Approved
Proguanil	--	Unknown. (metabolite is cycloguanil)	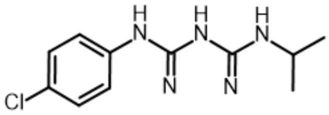	Approved
Pyrimethamine	--	PfDHFR	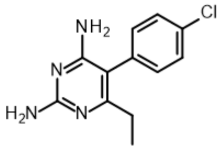	Approved
Pyronaridine	Tetraphosphate	haemozoin formation	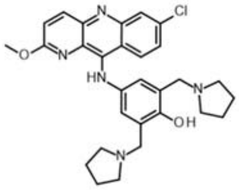	Approved
Quinine	Sulfate (quinine dimer)	haemoglobin digestion	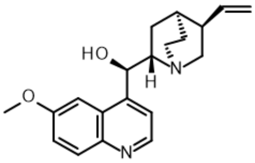	Approved
(+)-SJ733	Base or Hydrochloride	PfATP4	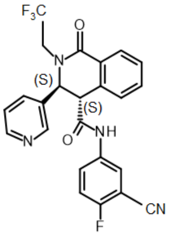	Phase II

Sulfadoxine	--	PfDHPS	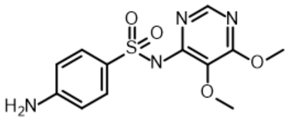	Approved
Sulfamethox-azole	--	PfDHPS	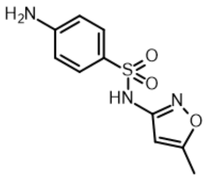	Approved
Tafenoquine	Base or Succinate	not known	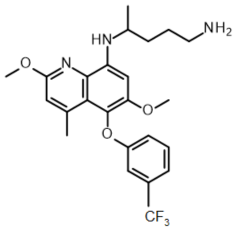	FDA Approved 2018
TDD-E209	Mesylate	not known (likely poly-pharmacology)	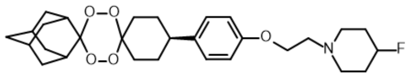	Preclinical

Table S2 Representative analytical conditions for quantitative analysis

Compound	Column	Mobile Phase / Flow rate (mL/min)	Gradient	Detection
Amodiaquine	Ascentis Express Amide (50 x 2.1 mm, 2.7 µm)	ACN/water (0.05% formic acid) / 0.4	0-60% ACN over 4 min	ESI positive MRM; m/z 356.18 > 283.0; Cone 35 V; CID 25 V [a]
AQ-13	Ascentis Express Amide (50 x 2.1 mm, 2.7 µm)	MeOH/water (2.5 mM ammonium formate, 5 mM formic acid) / 0.4	20-95% Methanol over 4 min	ESI positive MRM; m/z 292.23 > 113.96; Cone 35 V; CID 20 V [a]
Artemether	Ascentis Express Amide (50 x 2.1 mm, 2.7 µm)	ACN/water (0.05% formic acid) / 0.4	2-95% ACN over 4 min	ESI positive MRM; m/z 267.08 > 162.85; Cone 40 V; CID 45 V
Artemisone	Ascentis Express Amide (50 x 2.1 mm, 2.7 µm)	ACN/water (0.05% formic acid) / 0.4	20-95% ACN over 4 min	ESI positive MRM; m/z 402.45 > 163.15; Cone 30 V; CID 18 V
Artesunate	Ascentis Express Amide (50 x 2.1 mm, 2.7 µm)	ACN/water (0.05% formic acid) / 0.4	10-95% ACN over 4 min	ESI positive MRM; m/z 267.21 > 163.11; Cone 30 V; CID 7 V
Atovaquone	Ascentis Express Amide (50 x 2.1 mm, 2.7 µm)	ACN/water (5 mM ammonium formate) / 0.4	60-95% ACN over 4 min	ESI positive MRM; m/z 365.02 > 336.89; Cone 50 V; CID 30 V
Azithromycin	Ascentis Express Amide (50 x 2.1 mm, 2.7 µm)	MeOH/water (5 mM ammonium formate) / 0.4	2-95% Methanol over 4 min	ESI positive MRM; m/z 749.56 > 116.03; Cone 45 V; CID 40 V [a]
Chloroquine	Ascentis Express Amide (50 x 2.1 mm, 2.7 µm)	ACN/water (0.05% formic acid) / 0.4	0-70% ACN over 4 min	ESI positive MRM; m/z 320.24 > 247.14; Cone 35 V; CID 20 V
Chlorproguanil	Ascentis Express Amide (50 x 2.1 mm, 2.7 µm)	ACN/water (0.05% formic acid) / 0.4	10-80% ACN over 4 min	ESI positive MRM; m/z 288.15 > 203.97; Cone 30 V; CID 18 V
Clindamycin	Ascentis Express Amide (50 x 2.1 mm, 2.7 µm)	ACN/water (0.05% formic acid) / 0.4	5-60% ACN over 4 min	ESI positive MRM; m/z 425.07 > 125.93; Cone 30 V; CID 30 V [a]
Cycloguanil	Ascentis Express Amide (50 x 2.1 mm, 2.7 µm)	ACN/water (0.05% formic acid) / 0.4	5-60% ACN over 4 min	ESI positive MRM; m/z 252.31 > 195.03; Cone 30 V; CID 20 V [a]
Dapsone	Ascentis Express Amide (50 x 2.1 mm, 2.7 µm)	ACN/water (0.05% formic acid) / 0.4	10-80% ACN over 4 min	ESI positive MRM; m/z 249.20 > 155.95; Cone 30 V; CID 15 V
Dihydroartemisinin	Ascentis Express Amide (50 x 2.1 mm, 2.7 µm)	ACN/water (0.05% formic acid) / 0.4	2-95% ACN over 4 min	ESI positive MRM; m/z 267.15 > 163.24; Cone 40 V; CID 45 V
Doxycyclin	Ascentis Express Amide (50 x 2.1 mm, 2.7 µm)	ACN/water (0.05% formic acid) / 0.4	10-80% ACN over 4 min	ESI positive MRM; m/z 445.23 > 428.08; Cone 30 V; CID 18 V [a]
DSM265	Ascentis Express Amide (50 x 2.1 mm, 2.7 µm)	ACN/water (0.05% formic acid) / 0.4	10-70% ACN over 4 min	ESI positive MRM; m/z 416.05 > 152.82; Cone 45 V; CID 35 V
DSM421	Ascentis Express Amide (50 x 2.1 mm, 2.7 µm)	ACN/water (0.05% formic acid) / 0.4	10-95% ACN over 4 min	ESI positive MRM; m/z 359.56 > 153.37; Cone 40 V; CID 30 V

**Compound**	Column	Mobile Phase / Flow rate (mL/min)	Gradient	Detection
ELQ300	Ascentis Express Amide (50 x 2.1 mm, 2.7 µm)	ACN/water (0.05% formic acid) / 0.4	10-95% ACN over 4 min	ESI positive MRM; m/z 476.10 > 433.0; Cone 50 V; CID 40 V
Ferroquine	Ascentis Express Amide (50 x 2.1 mm, 2.7 µm)	ACN/water (0.05% formic acid) / 0.4	2-95% ACN over 4 min	ESI positive MRM; m/z 434.0 > 389.0; Cone 25 V; CID 20 V
Halofantrine	Ascentis Express Amide (50 x 2.1 mm, 2.7 µm)	ACN/water (0.05% formic acid) / 0.4	10-70% ACN over 4 min	ESI positive MRM; m/z 500.22 > 142.18; Cone 40 V; CID 25 V [a]
JPC3210	Ascentis Express Amide (50 x 2.1 mm, 2.7 µm)	ACN/water (0.05% formic acid) / 0.4	5-70% ACN over 4 min	ESI positive MRM; m/z 399.24 > 326.25; Cone 40 V; CID 25 V [a]
KAE609	Ascentis Express Amide (50 x 2.1 mm, 2.7 µm)	ACN/water (0.05% formic acid) / 0.4	0-70% ACN over 4 min	ESI positive MRM; m/z 390.18 > 347.02; Cone 30 V; CID 15 V [a]
KAF156	Ascentis Express Amide (50 x 2.1 mm, 2.7 µm)	ACN/water (0.05% formic acid) / 0.4	10-70% ACN over 4 min	ESI positive MRM; m/z 412.24 > 100.80; Cone 40 V; CID 25 V [a]
Lumefantrine	Ascentis Express Amide (50 x 2.1 mm, 2.7 µm)	ACN/water (0.05% formic acid) / 0.4	10-70% ACN over 4 min	ESI positive MRM; m/z 528.01 > 510.12; Cone 40 V; CID 25 V
M5717	Ascentis Express Amide (50 x 2.1 mm, 2.7 µm)	MeOH/water (5 mM ammonium formate) / 0.4	5-95% Methanol over 4 min	ESI positive MRM; m/z 463.37 > 392.24; Cone 45 V; CID 30 V [a]
Mefloquine	Ascentis Express Amide (50 x 2.1 mm, 2.7 µm)	ACN/water (0.05% formic acid) / 0.4	10-60% ACN over 4 min	ESI positive MRM; m/z 379.10 > 361.20; Cone 40 V; CID 35 V
MMV048	Ascentis Express Amide (50 x 2.1 mm, 2.7 µm)	ACN/water (0.05% formic acid) / 0.4	10-70% ACN over 4 min	ESI positive MRM; m/z 393.96 > 315.12; Cone 45 V; CID 35 V
MMV052	Ascentis Express Amide (50 x 2.1 mm, 2.7 µm)	ACN/water (0.05% formic acid) / 0.4	5-95% ACN over 4 min	ESI positive MRM; m/z 526.42 > 360.26; Cone 35 V; CID 40 V [a]
MMV253	Ascentis Express Amide (50 x 2.1 mm, 2.7 µm)	ACN/water (0.05% formic acid) / 0.4	5-80% ACN over 4 min	ESI positive MRM; m/z 466.09 > 395.05; Cone 40 V; CID 29 V [a]
Naphthoquine	Ascentis Express Amide (50 x 2.1 mm, 2.7 µm)	ACN/water (0.05% formic acid) / 0.4	0-60% ACN over 4 min	ESI positive MRM; m/z 410.16 > 337.18; Cone 50 V; CID 25 V [a]
N-Desethylamodiaquine	Ascentis Express Amide (50 x 2.1 mm, 2.7 µm)	ACN/water (0.05% formic acid) / 0.4	0-95% ACN over 4 min	ESI positive MRM; m/z 328.07 > 283.09; Cone 30 V; CID 15 V [a]
NPC1161B	Ascentis Express Amide (50 x 2.1 mm, 2.7 µm)	ACN/water (0.05% formic acid) / 0.4	20-95% ACN over 4 min	ESI positive MRM; m/z 434.10 > 86.16; Cone 30 V; CID 20 V
OZ277	Ascentis Express Amide (50 x 2.1 mm, 2.7 µm)	ACN/water (0.05% formic acid) / 0.4	0-95% ACN over 4 min	ESI positive MRM; m/z 393.28 > 227.22; Cone 25 V; CID 15 V
OZ439	Ascentis Express Amide (50 x 2.1 mm, 2.7 µm)	ACN/water (0.05% formic acid) / 0.4	2-95% ACN over 4 min	ESI positive MRM; m/z 470.21 > 303.88; Cone 35 V; CID 35 V
P218	Ascentis Express Amide (50 x 2.1 mm, 2.7 µm)	ACN/water (0.05% formic acid) / 0.4	5-60% ACN over 4 min	ESI positive MRM; m/z 361.10 > 155.05; Cone 45 V; CID 40 V

Compound	Column	Mobile Phase / Flow rate (mL/min)	Gradient	Detection
Piperaquine	Ascentis Express Amide (50 x 2.1 mm, 2.7 µm)	ACN/water (0.05% formic acid) / 0.4	0-95% ACN over 4 min	ESI positive MRM; m/z 535.15 > 288.11; Cone 45 V; CID 35 V [a]
Primaquine	Ascentis Express Amide (50 x 2.1 mm, 2.7 µm)	ACN/water (0.05% formic acid) / 0.4	10-70% ACN over 4 min	ESI positive MRM; m/z 260.28 > 85.96; Cone 25 V; CID 15 V
Proguanil	Phenomenex Synergi Polar (50 x 2.0 mm, 2.5 µm)	ACN/water (0.05% formic acid) / 0.4	0-95% ACN over 4 min	ESI positive MRM; m/z 254.20 > 102.04; Cone 40 V; CID 25 V
Pyrimethamine	Ascentis Express Amide (50 x 2.1 mm, 2.7 µm)	ACN/water (0.05% formic acid) / 0.4	10-80% ACN over 4 min	ESI positive MRM; m/z 249.27 > 176.95; Cone 30 V; CID 30 V
Pyronaridine	Ascentis Express Amide (50 x 2.1 mm, 2.7 µm)	ACN/water (0.05% formic acid) / 0.6	0-95% ACN over 4 min	ESI positive MRM; m/z 518.22 > 447.13; Cone 30 V; CID 20 V [a]
Quinine	Ascentis Express Amide (50 x 2.1 mm, 2.7 µm)	ACN/water (0.05% formic acid) / 0.4	5-60% ACN over 4 min	ESI positive MRM; m/z 325.25 > 81.04; Cone 30 V; CID 30 V [a]
SJ733	Ascentis Express Amide (50 x 2.1 mm, 2.7 µm)	ACN/water (0.05% formic acid) / 0.4	2-95% ACN over 4 min	ESI positive MRM; m/z 468.97 > 189.04; Cone 40 V; CID 30 V [a]
Sulfadoxine	Ascentis Express Amide (50 x 2.1 mm, 2.7 µm)	ACN/water (0.05% formic acid) / 0.4	10-70% ACN over 4 min	ESI positive MRM; m/z 311.15 > 155.89; Cone 35 V; CID 16 V
Sulfamethoxazole	Ascentis Express Amide (50 x 2.1 mm, 2.7 µm)	ACN/water (0.05% formic acid) / 0.4	10-80% ACN over 4 min	ESI positive MRM; m/z 254.13 > 155.89; Cone 30 V; CID 15 V
Tafenoquine	Ascentis Express Amide (50 x 2.1 mm, 2.7 µm)	ACN/water (0.05% formic acid) / 0.4	10-70% ACN over 4 min	ESI positive MRM; m/z 464.28 > 85.90; Cone 35 V; CID 20 V [a]
TDD-E209	Ascentis Express Amide (50 x 2.1 mm, 2.7 µm)	ACN/water (0.05% formic acid) / 0.4	2-95% ACN over 4 min	ESI positive MRM; m/z 502.21 > 130.06; Cone 40 V; CID 25 V [a]

[a] metabolism samples analyzed by ESI positive MS^E with a cone voltage of 30-35 V

Table S3 Representative assay validation data for quantitative methods. Calibration standards were prepared in the same matrix as the study samples for all assays.

Compound	Accuracy	Rel. S.D.	Recovery [a]	LLQ range (ng/mL)
Amodiaquine	± 6%	< 7%	> 90%	1-5
AQ-13	± 6%	< 8%	> 85%	1-5
Artemether	± 7%	< 10%	> 99%	10-50
Artemisone	± 13%	< 5%	> 90%	5
Artesunate	± 11%	< 11%	> 95%	2.5-5
Atovaquone	± 13%	< 12%	> 90%	2.5-5
Azithromycin	± 15%	< 13%	> 85%	1-5
Chloroquine	± 10%	< 12%	> 95%	1-5
Chlorproguanil	± 7%	< 8%	> 80%	1
Clindamycin	± 12%	< 6%	> 90%	1
Cycloguanil	± 10%	< 10%	> 95%	1
Dapsone	± 9%	< 7%	> 90%	1-5
Dihydroartemisinin	± 7%	< 12%	> 99%	2.5-5
Doxycyclin	± 7%	< 13%	> 90%	1-5
DSM265	± 12%	< 12%	> 80%	1-5
DSM421	± 9%	< 8%	> 99%	10
ELQ300	± 13%	< 13%	> 85%	0.5-5
Ferroquine	± 7%	< 10%	> 90%	1-5
Halofantrine	± 11%	< 10%	> 95%	0.5-5
JPC3210	± 6%	< 9%	> 95%	0.5-5
KAE609	± 9%	< 13%	> 95%	5-10
KAF156	± 15%	< 14%	> 94%	5-10
Lumefantrine	± 13%	< 10%	> 95%	2-5
M5717	± 11%	< 6%	> 90%	5
Mefloquine	± 6%	< 8%	> 80%	1-5
MMV048	± 7%	< 9%	> 90%	1-5
MMV052	± 7%	< 6%	> 90%	1
MMV253	± 14%	< 12%	> 95%	5-10
N-Desethylamodiaquine	± 10%	< 12%	> 85%	10-50
Naphthoquine	± 12%	< 10%	> 70%	1
NPC1161B	± 10%	< 7%	> 95%	2-10
OZ277	± 6%	< 7%	> 50%	1
OZ439	± 11%	< 8%	> 95%	1
P218	± 12%	< 3%	> 80%	1-5
Piperaquine	± 6%	< 12%	> 95%	5-50
Primaquine	± 11%	< 12%	> 70%	1-5
Proguanil	± 6%	< 7%	> 90%	1-2.5
Pyrimethamine	± 12%	< 7%	> 80%	1-5
Pyronaridine	± 6%	< 16%	> 90%	50-100
Quinine	± 8%	< 13%	> 90%	20-50
Sulfadoxine	± 6%	< 15%	> 80%	1
Sulfamethoxazole	± 8%	< 7%	> 85%	1-10
SJ733	± 6%	< 6%	> 95%	0.5-1
Tafenoquine	± 8%	< 11%	> 99%	2.5-10
TDD-E209	± 11%	< 13%	> 95%	1-5

[a] Recovery data are typically for assays using plasma or mixed matrices (e.g. plasma/buffer or plasma/whole blood)

Table S4 Metabolic pathways and positive control inhibitors. Data for K_m represent the fitted values (S.E. of the estimate).

CYP isoform	Microsomal protein (mg/mL)	Incubation time (min)	Substrate (concentration)	Metabolic pathway	Measured K_m (µM)[a]	Literature K_m (µM) (range)	Positive control inhibitor
CYP1A2	0.1	30	phenacetin (40 µM)	phenacetin O-deethylation	57.6 (4.6)	47 [1] (9 – 68) [b]	furafylline
CYP2C9	0.2	30	tolbutamide (140 µM)	tolbutamide methylhydroxylation	371 (13)	147 [1] (60 – 580) [b]	sulfaphenazole
CYP2C19	0.2	40	(S)-mephenytoin (30 µM)	(S)-mephenytoin 4'-hydroxylation	44.0 (4.1)	57.2 [1] (23 – 169) [b]	ticlopidine
CYP2D6	0.1	15	dextromethorphan (2 µM)	dextromethorphan O-demethylation	3.28 (0.51)	4.64 [1] (2.8 – 22) [b]	quinidine
CYP3A4	0.1	10	midazolam (1.25 µM)	midazolam 1'-hydroxylation	1.82 (0.12)	2.27 [1] (2.4 – 12) [b]	ketoconazole
CYP3A4	0.1	10	testosterone (20 µM)	testosterone 6β-hydroxylation	49.7 (10)	46.4 [1] (31 – 206) [b]	ketoconazole

[a] K_m values for each CYP-specific biotransformation were determined under the same incubation conditions as was used for the assessment of CYP inhibition (see Methods, main text). Initial rates of metabolite formation (confirmed based on linearity of product formation with respect to time) were assessed over a range of substrate concentrations spanning approximately 10-fold below to >6-fold above the anticipated K_m. Enzyme kinetic parameters were determined by fitting the standard Michaelis-Menten model using GraphPad Prism (v 7) software.

[b] [1] and references therein

Table S5 Analytical conditions used for CYP inhibition studies

**Metabolite**	Column	Mobile Phase / Flow rate (mL/min)	Gradient	Detection
paracetamol	Ascentis Express Amide (50 x 2.1 mm, 2.7 µm)	ACN/water (0.05% formic acid) / 0.6	0-70% ACN over 2.5 min	ESI positive MRM; m/z 152.08 > 110.04; Cone 35 V; CID 25 V
methylhydroxy-tolbutamide	Ascentis Express Amide (50 x 2.1 mm, 2.7 µm)	ACN/water (0.05% formic acid) / 0.6	0-70% ACN over 2.5 min	ESI positive MRM; m/z 287.03 > 74.05; Cone 35 V; CID 25 V
4'-Hydroxy- mephenytoin	Ascentis Express Amide (50 x 2.1 mm, 2.7 µm)	ACN/water (0.05% formic acid) / 0.6	0-70% ACN over 2.5 min	ESI positive MRM; m/z 235.10 > 150.22; Cone 40 V; CID 25 V
dextrorphan	Ascentis Express Amide (50 x 2.1 mm, 2.7 µm)	ACN/water (0.05% formic acid) / 0.6	0-70% ACN over 2.5 min	ESI positive MRM; m/z 257.90 > 157.07; Cone 50 V; CID 50 V
1-hydroxy-midazolam	Ascentis Express Amide (50 x 2.1 mm, 2.7 µm)	ACN/water (0.05% formic acid) / 0.6	0-70% ACN over 2.5 min	ESI positive MRM; m/z 341.83 > 324.07; Cone 50 V; CID 35 V
6β-hydroxy- testosterone	Ascentis Express Amide (50 x 2.1 mm, 2.7 µm)	ACN/water (0.05% formic acid) / 0.6	0-70% ACN over 2.5 min	ESI positive MRM; m/z 305.12 > 269.10; Cone 40 V; CID 15 V

Table S6 Calculated pKa and Log D values using ChemAxon or ACD Labs

Compound	calculated pKa ChemAxon / ACD	Calculated Log $D_{7.4}$ ChemAxon / ACD [a]
Amodiaquine	10.2 (B), 6.5 (B), 9.1 (A) / 5.6 (B), 9.4 (A)	2.32 / 0.95
AQ-13	9.9 (B), 7.3 (B) / 10.3 (B)	0.26 / 0.91
Artemether	-- / --	3.48 / 2.82
Artemisone	2.1 (B) / 2.99	2.21 / 2.89
Artesunate	3.8 (A) / 4.3 (A)	-0.17 / 0.26
Atovaquone	8.2 (A) / 5.0 (A)	3.33 / 4.11
Azithromycin	8.7 (B), 7.6 (B) / 8.6 (B)	-1.23 / 0.66
Chloroquine	9.6 (B), 8.9 (B) / 10.5 (B)	0.88 / 1.59
Chlorproguanil	10.0 (B), 7.7 (B) / 10.8 (B)	0.31 / 2.12
Clindamycin	7.6 (B) / 8.7 (B)	0.65 / 0.70
Cycloguanil	8.2 (B), 5.9 (B) / 9.3 (B)	0.86 / -0.69
Dapsone	2.4 (B) / 1.2 (B)	1.27 / 0.99
Dihydroartemisinin	-- / --	2.84 / 2.19
Doxycyclin	7.8 (B), 4.7 (A) / 10.8 (B), 4.5 (A)	-3.57 / -1.1
DSM265	<2 / 2.4 (B)	5.68 / 2.63
DSM421	<2 / NA	3.34 / NA
ELQ300	-- / --	7.20 / 6.2
Ferroquine	9.0 (B), 7.3 (B) / NA	1.72 / NA
Halofantrine	10.1 (B) / 9.4 (B)	5.46 / 6.93
JPC3210	10.7 (B), 7.9 (A) / NA	3.96 / NA
KAE609	6.5 (B) / 6.1 (B)	4.11 / 4.11
KAF156	8.1 (B), 5.7 (B) / 8.0 (B)	2.26 / 2.40
Lumefantrine	9.8 (B) / 8.7 (B)	6.84 / 7.42
M5717	8.5 (B), 7.1 (B) / NA	2.28 /NA
Mefloquine	9.5 (B) / 9.2 (B)	2.07 / 0.50
MMV048	5.9 (B) / 3.8 (B)	2.69 / 0.64
MMV052	10.1 (B) / NA	3.35 / NA
MMV253	7.5 (B), 4.4 (B), 3.4 (B), 11.9 (A) / NA	3.86 / NA
N-Desethylamodiaquine	10.6 (B), 6.5 (B), 9.2 (A) / NA	1.43 /NA
Naphthoquine	10.8 (B), 6.5 (B), 9.6 (A) / NA	3.35 / NA
NPC1161B	10.5 (B), 5.0 (B) / 10.4 (B)	2.26 / 2.67
OZ277	9.6 (B) / 9.1 (B)	0.94 / 0.15
OZ439	6.7 (B) / 6.5 (B)	5.36 / 4.37
P218	7.6 (B), 4.3 (A) / 7.0 (B), 4.7 (A)	-0.20 / -1.32
Piperaquine	8.8 (B), 7.4 (B), 6.8 (B) / 8.9 (B)	3.87 / 5.63
Primaquine	10.2 (B), 4.1 (B) / 10.4 (B)	-0.96 / 0.01
Proguanil	10.4 (B), 8.2 (B) / 11.1 (B)	-0.78 / 1.13
Pyrimethamine	7.8 (B) /7.2 (B)	2.23 / 2.55
Pyronaridine	10.2 (B), 9.2 (B), 5.9 (B), 8.0 (A) / 10.2 (B), 8.2 (A)	2.08 / 1.15
Quinine	9.0 (B), 4.0 (B) / 9.3 (B)	0.86 / 0.98
SJ733	4.8 (B) / NA	3.64 / NA
Sulfadoxine	2.6 (B), 6.10 (A) / 2.2 (B), 6.2 (A)	-0.22 / -1.38
Sulfamethoxazole	6.2 (A) / 5.8 (A)	0.00 / -0.54
Tafenoquine	10.2 (B), 3.2 (B) / 10.4 (B)	2.37 / 3.09
TDD-E209	8.4 (B) / NA	4.99 / NA

[a] ACD values were obtained from ChEMBL (https://www.ebi.ac.uk/chembl/)

Table S7 Permeability data for control compounds across Caco-2 cell monolayers over the course of the study.

Compound	P_{app} (10^{-6} cm/s) [a]		Efflux Ratio Range
	A – B Range	B – A Range	
Lucifer Yellow	<2	Not assessed	--
Propranolol	29 – 41	35 – 42[c]	0.9 – 1.0
Rhodamine 123	0.7 - 1.6	10 – 28	6 – 39
Compound	P_{app} (10^{-6} cm/s) [b]		Efflux Ratio Range
	A – B Range	B – A Range	
Lucifer Yellow	<3	Not assessed	--
Propranolol	78 – 121	81 – 107[c]	0.9 – 1.4
Rhodamine 123	0.6 – 1.7	9 – 31	11 – 21

[a] pH 7.4 buffer as medium
[b] plasma as transport medium
[c] B-A P_{app} for propranolol not assessed in all experiments

Table S8 Fraction unbound in human plasma for control compounds using the different methods. Data represent the mean (S.D.) for 3-4 technical replicates (UC) or 3-4 individual dialysis units (RED).

Compound (Log $D_{7.4}$)	Log $D_{7.4}$	f_u – UC (neat plasma)	f_u – RED (neat plasma, 6 h incubation)	f_u – RED (10% plasma; 6 h dialysis)	f_u – RED (10% plasma; 24 h dialysis with presaturation)	Literature f_u, Method
Propranolol	0.36	0.29 (0.016)	0.32 (0.0096)	0.29 (0.082)	0.17 (0.0038)	0.101 (0.013), UC [2]<br>0.231 (0.046), RED [3]
(RS)-ketoprofen	0.39	0.010 (0.001)	0.0074 (0.0001) [a]	0.012 (0.001) [a]	0.0048 (0.0004)	0.013 (0.002), ED [4]
warfarin	0.94	0.010 (0.001)	0.012 (0.001)<br>0.0060 (0.0011) [a]	0.013 (0.001)<br>0.0047 (0.0002)	0.0047 (n=2) [a]	0.010 (0.001), UC [2]<br>0.014 (0.003), RED [3]<br>0.015 (0.003) (R), ED [5]<br>0.012 (0.002) (S), ED [5]
Saquinavir	2.05	0.053 (0.011) [b]	0.0069 (0.00080) [a]	0.0077 (0.0007) [a]	0.0077 (0.0007)	0.014 (0.001), ED [5]
verapamil	2.79	0.200 (0.009)	0.130 (0.011) [c]<br>0.092 (0.0013) [a]	0.140 (0.011) [c]	0.085 (0.012) [a]	0.123 (0.0003), UC [2]<br>0.160 (0.016), RED [3]
ketoconazole	4.13	0.018 (0.001)	0.0072 (0.0009)	0.011 (0.0005)	0.0066 (0.0013)	0.018 (0.001), ED [5]

UC=ultracentrifugation
RED=rapid equilibrium dialysis
ED=equilibrium dialysis
[a,c] same batches of plasma used for these measurements
[b] likely an overestimation of the fraction unbound

Table S9 **Fraction unbound in human plasma for antimalarial compounds using different methods (where assessed). Data represent the mean (S.D.) for 3-4 technical replicates; duplicate values represent independent experiments.**

Compound	f_u – UC (neat plasma)	f_u – RED (10% plasma; 6 h dialysis)	f_u – RED (10% plasma; 24 h dialysis with presaturation)	Literature f_u, Method
Amodiaquine	0.089 (0.0080)	0.086 (0.0027)	Not assessed	<0.1, ND [6]
AQ-13	0.43 (0.022)	Not assessed	Not assessed	0.45, UF [7]
Artemether	0.10 (0.018)	Not assessed	0.038 (0.0090)<br>0.066 (0.0090)	0.046, EP [8]
Atovaquone	0.0020 (0.00020)	<0.0002	<0.0001	0.001 [9]
Azithromycin	0.71 (0.059)	0.34, 0.35	Not assessed	0.5-0.88, ED [10]
Chloroquine	0.54 (0.048)<br>0.61 (0.023)	Not assessed	Not assessed	0.54, ED [11]
Clindamycin	0.19 (0.011)	Not assessed	Not assessed	0.06, UF [12]
Dapsone	0.27 (0.019)	Not assessed	Not assessed	0.26, UF [13]
Dihydroartemisinin	0.21 (0.010)	Not assessed	Not assessed	0.092, UF [14]
Doxycyclin	0.23 (0.090)	Not assessed	Not assessed	0.12, ED [15]
DSM265	0.0080 (0.0010)	0.0020 (0.0001)	0.0018 (0.00010)	Not available
DSM421	0.039 (0.0070)	0.035 (0.0044)	Not assessed	Not available
ELQ300	0.011 (0.0020)	<0.0001	0.00010 (0.000010)	Not available
Ferroquine	0.070 (0.0080)	Not assessed	0.041 (0.0010)	Not available
Halofantrine	0.13 (0.032)	<0.0001	<0.0001	Not available
JPC3210	0.071 (n=2)	0.0011 (0.0002)	0.0028 (0.00020)<br>0.0020 (0.00010)	0.024, UF [16]
KAE609	0.057 (0.0050)	0.0020 (0.0002)	0.0016 (0.00010)	Not available
KAF156	0.11 (0.0080)	0.069 (0.013)	Not assessed	Not available
Lumefantrine	not assessed	<0.0001	<0.0001	0.003, EP [8]
M5717	0.34 (0.021)	0.24 (0.0020)	Not assessed	0.23, ED [17]
Mefloquine	0.058 (0.0020)	0.015 (0.0010)	Not assessed	0.02, ND [18]
MMV048	0.17 (0.0050)	0.110 (0.0070)	Not assessed	Not available
MMV052	0.058 (0.011)	<0.0001	0.00040 (0.00010)<br>0.00023 (0.00003)	Not available
MMV253	0.067 (0.0050)	Not assessed	0.017 (0.00020)	Not available
N -Desethylamodiaquine	0.17 (0.0020)	Not assessed	Not assessed	0.18, UF [16]
Naphthoquine	0.030 (0.0020)	0.019 (0.0010)	0.018 (0.0010)	Not available
NPC1161B	0.046 (0.0090)	<0.0003	0.00074 (0.00016)	Not available
OZ277	0.086 (0.0080)	0.056 (0.0037)	Not assessed	Not available
OZ439	0.099 (0.024)	<0.0001	<0.0001	Not available
Piperaquine	0.042 (0.0060)	<0.001	0.0003 (0.0001)	<0.001, ND [19]
Primaquine	0.26 (0.023)	Not assessed	Not assessed	0.35-0.55, ND [9]
Proguanil	0.34 (0.010)	Not assessed	Not assessed	0.25, ND [9]
Pyrimethamine	0.085 (0.0060)	0.095 (0.0010)	Not assessed	0.088, ED [20]
Pyronaridine	0.21 (0.011)	0.090 (0.010)	Not assessed	<0.35, ND [9]
Quinine	0.20 (0.013)	Not assessed	Not assessed	0.15, ED [21]
SJ733	0.17 (0.0050)	0.10 (0.024)	0.085 (0.012)	Not available
Sulfadoxine	0.036 (0.0010)	0.025 (0.0011)	Not assessed	0.05-0.1, ND [9]
Sulfamethoxazole	0.32 (0.024)	Not assessed	Not assessed	0.27, ED [22]
Tafenoquine	0.11 (0.015)	<0.0007	0.00070 (0.00017)	<0.005, UF [16]
TDD-E209	0.094 (0.011)	<0.0001	<0.0001	Not available

UF=ultrafiltration
ED=equilibrium dialysis
EP=erythrocyte partitioning method
ND=method not described

Table S10 ***In vitro*** **intrinsic clearance for control compounds. Data represent the mean (S.D.) for 11-21 independent measurements except for midazolam which was only 6 independent measurements over the course of the study. Literature values were obtained using a similar microsomal protein concentration (0.5 mg/mL) compared to our studies (0.4 mg/mL).**

Compound	Range HLM CL_{int} (µL/min/mg)	Mean CL_{int} (µL/min/mg)	Literature CL_{int} (µL/min/mg)
omeprazole	15.9 – 40.9	24.2 (8.5)	18 [23], 78.3 [24]
phenacetin	13.3 – 31.1	21.4 (6.4)	27 [23], 22.2 [24]
dextromethorphan	20.4 – 55.4	37.3 (10.1)	23 [25], 62 [26]
verapamil	69 – 185	118 (30)	142 [23], 140 [24], 139 [27], 153 [28]
diclofenac	107 – 391	183 (67)	207[23], 210 [27], 294 [28]
midazolam	240 – 300	272 (22)	353 [23], 178 [27], 287 [28]

Table S11 Measured IC_{50} and K_i values for positive control inhibitors. Data for K_i represent mean (S.D.) for 6-9 independent measurements over the course of the study.

CYP isoform	Metabolic pathway	Positive control inhibitor	IC_{50} (μM)	K_i (μM)	Literature K_i (μM) (range)
CYP1A2	phenacetin O-deethylation	furafylline[c]	4.47 (0.60)	2.64 (0.35)	0.88 [1] [a], (0.6-4.4) [29] [b]
CYP2C9	tolbutamide methylhydroxylation	sulfaphenazole	0.633 (0.15)	0.459 (0.13)	0.135 [1] [a], (0.12-0.7) [29] [b]
CYP2C19	(S)-mephenytoin 4'-hydroxylation	ticlopidine[c]	2.35 (0.82)	1.40 (0.49)	1.2 [30], (0.02-3.7) [29] [b]
CYP2D6	dextromethorphan O-demethylation	quinidine	0.0220 (0.0059)	0.0162 (0.0079)	0.029 [1] [a], (0.03-0.4) [29] [b]
CYP3A4	midazolam 1'-hydroxylation	ketoconazole	0.0162 (0.0039)	0.00973 (0.0023)	0.0095 [1] [a], (0.004-0.18) [31] [b]
CYP3A4	testosterone 6β-hydroxylation	ketoconazole	0.0154 (0.0067)	0.0110 (0.0048)	0.0130 [1] [a], (0.045-0.3) [31] [b]

[a] calculated from IC_{50} / 2 assuming competitive inhibition; substrate concentration used by authors was equal to the K_m
[b] and references therein
[c] mechanism-based inhibitor [32]

Table S12 Comparison of measured Caco-2 P_{app} value with data from the literature.

Compound	A-B P_{app} (10^{-6} cm/sec)	ER	Literature P_{app} (10^{-6} cm/sec), ER
Amodiaquine	90 ± 8.3 [a]	0.7	16.0 ± 1.1, 1.3 [33]
Artemisone	58.8, 64.5	0.8	59.6 ± 4.2, 0.8 [33]
Artesunate	3.4, 2.9	0.9	10.2 ± 0.3, 1.2 [33],<br>4.0 ± 0.4 [34]
Dihydroartemisinin	50 ± 2.3	1.0	38.6 ± 5.0 [35]
Mefloquine	180 ± 20 [a]	0.83	9.9 ± 0.7, 0.7 [33]
Naphthoquine	230 ± 32 [a]	0.8	27.3 ± 0.6, 0.8 [35]
Pyronaridine	16 ± 2.2 [a]	2.6	10.3 ± 0.3, 1.8 [35]
Piperaquine	>300 [a]	---	0 [35]

[a] Caco-2 experiment conducted in the presence of human plasma to improve mass balance and sink conditions. The f_u value was taken into account when calculating the apparent P_{app} values.

REFERENCES

1. Walsky RL, Obach RS. Validated assays for human cytochrome P450 activities. Drug Metab Dispos 2004; 32:647-660.

2. Nakai D, Kumamoto K, Sakikawa C, Kosaka T, Tokui T. Evaluation of the protein binding ratio of drugs by a micro-scale ultracentrifugation method. J Pharm Sci 2004; 93:847-854.

3. Curran RE, Claxton CR, Hutchison L, Harradine PJ, Martin IJ, Littlewood P. Control and measurement of plasma pH in equilibrium dialysis: influence on drug plasma protein binding. Drug Metab Dispos 2011; 39:551-557.

4. Williams RL, Upton RA, Buskin JN, Jones RM. Ketoprofen-aspirin interactions. Clin Pharmacol Ther 1981; 30:226-231.

5. Kochansky CJ, McMasters DR, Lu P, Koeplinger KA, Kerr HH, Shou M, Korzekwa KR. Impact of pH on plasma protein binding in equilibrium dialysis. Mol Pharm 2008; 5:438-448.

6. Krishna S, White NJ. Pharmacokinetics of quinine, chloroquine and amodiaquine. Clinical implications. Clin Pharmacokinet 1996; 30:263-299.

7. Ramanathan-Girish S, Catz P, Creek MR, Wu B, Thomas D, Krogstad DJ, De D, Mirsalis JC, Green CE. Pharmacokinetics of the antimalarial drug, AQ-13, in rats and cynomolgus macaques. Int J Toxicol 2004; 23:179-189.

8. Colussi D, Parisot C, Legay F, Lefevre G. Binding of artemether and lumefantrine to plasma proteins and erythrocytes. Eur J Pharm Sci 1999; 9:9-16.

9. Giao PT, de Vries PJ. Pharmacokinetic interactions of antimalarial agents. Clin Pharmacokinet 2001; 40:343-373.

10. Foulds G, Shepard RM, Johnson RB. The pharmacokinetics of azithromycin in human serum and tissues. J Antimicrob Chemother 1990; 25 Suppl A:73-82.

11. Walker O, Birkett DJ, Alvan G, Gustafsson LL, Sjoqvist F. Characterization of chloroquine plasma protein binding in man. Br J Clin Pharmacol 1983; 15:375-377.

12. Gordon RC, Regamey C, Kirby WM. Serum protein binding of erythromycin, lincomycin, and clindamycin. J Pharm Sci 1973; 62:1074-1077.

13. Ahmad RA, Rogers HJ. Plasma and salivary pharmacokinetics of dapsone estimated by a thin layer chromatographic method. Eur J Clin Pharmacol 1980; 17:129-133.

14. Batty KT, Ilett KF, Davis TM. Protein binding and alpha : beta anomer ratio of dihydroartemisinin in vivo. Br J Clin Pharmacol 2004; 57:529-533.

15. Houin G, Brunner F, Nebout T, Cherfaoui M, Lagrue G, Tillement JP. The effects of chronic renal insufficiency on the pharmacokinetics of doxycycline in man. Br J Clin Pharmacol 1983; 16:245-252.

16. Birrell GW, Heffernan GD, Schiehser GA, Anderson J, Ager AL, Morales P, MacKenzie D, van Breda K, Chavchich M, Jacobus LR, et al. Characterization of the preclinical pharmacology of the new 2-aminomethylphenol, JPC-3210, for malaria treatment and prevention. Antimicrob Agents Chemother 2018; 62:e01335-01317.

17. Baragana B, Hallyburton I, Lee MC, Norcross NR, Grimaldi R, Otto TD, Proto WR, Blagborough AM, Meister S, Wirjanata G, et al. A novel multiple-stage antimalarial agent that inhibits protein synthesis. Nature 2015; 522:315-320.

18. White NJ. Clinical pharmacokinetics of antimalarial drugs. Clin Pharmacokinet 1985; 10:187-215.

19. Hoglund RM, Adam I, Hanpithakpong W, Ashton M, Lindegardh N, Day NP, White NJ, Nosten F, Tarning J. A population pharmacokinetic model of piperaquine in pregnant and non-pregnant women with uncomplicated Plasmodium falciparum malaria in Sudan. Malar J 2012; 11:398.

20. Rudy AC, Poynor WJ. Binding of pyrimethamine to human plasma proteins and erythrocytes. Pharm Res 1990; 7:1055-1060.

21. Wanwimolruk S, Denton JR. Plasma protein binding of quinine: binding to human serum albumin, alpha 1-acid glycoprotein and plasma from patients with malaria. J Pharm Pharmacol 1992; 44:806-811.

22. Wijkstrom A, Westerlund D. Plasma protein binding of sulphadiazine, sulphamethoxazole and trimethoprim determined by ultrafiltration. J Pharm Biomed Anal 1983; 1:293-299.

23. Sohlenius-Sternbeck AK, Afzelius L, Prusis P, Neelissen J, Hoogstraate J, Johansson J, Floby E, Bengtsson A, Gissberg O, Sternbeck J, Petersson C. Evaluation of the human prediction of clearance from hepatocyte and microsome intrinsic clearance for 52 drug compounds. Xenobiotica 2010; 40:637-649.

24. Ito K, Houston JB. Prediction of human drug clearance from in vitro and preclinical data using physiologically based and empirical approaches. Pharm Res 2005; 22:103-112.

25. Youdim K, Dodia R. Comparison between recombinant P450s and human liver microsomes in the determination of cytochrome P450 Michaelis-Menten constants. Xenobiotica 2010; 40:235-244.

26. Chen Y, Liu L, Nguyen K, Fretland AJ. Utility of intersystem extrapolation factors in early reaction phenotyping and the quantitative extrapolation of human liver microsomal intrinsic clearance using recombinant cytochromes P450. Drug Metab Dispos 2011; 39:373-382.

27. Obach RS. Prediction of human clearance of twenty-nine drugs from hepatic microsomal intrinsic clearance data: An examination of in vitro half-life approach and nonspecific binding to microsomes. Drug Metab Dispos 1999; 27:1350-1359.

28. Giuliano C, Jairaj M, Zafiu CM, Laufer R. Direct determination of unbound intrinsic drug clearance in the microsomal stability assay. Drug Metab Dispos 2005; 33:1319-1324.

29. Khojasteh SC, Prabhu S, Kenny JR, Halladay JS, Lu AY. Chemical inhibitors of cytochrome P450 isoforms in human liver microsomes: a re-evaluation of P450 isoform selectivity. Eur J Drug Metab Pharmacokinet 2011; 36:1-16.

30. Ko JW, Desta Z, Soukhova NV, Tracy T, Flockhart DA. In vitro inhibition of the cytochrome P450 (CYP450) system by the antiplatelet drug ticlopidine: potent effect on CYP2C19 and CYP2D6. Br J Clin Pharmacol 2000; 49:343-351.

31. Greenblatt DJ, Venkatakrishnan K, Harmatz JS, Parent SJ, von Moltke LL. Sources of variability in ketoconazole inhibition of human cytochrome P450 3A in vitro. Xenobiotica 2010; 40:713-720.

32. Obach RS, Walsky RL, Venkatakrishnan K. Mechanism-based inactivation of human cytochrome p450 enzymes and the prediction of drug-drug interactions. Drug Metab Dispos 2007; 35:246-255.

33. Senarathna SM, Page-Sharp M, Crowe A. The interactions of P-Glycoprotein with antimalarial drugs, including substrate affinity, inhibition and regulation. PLoS One 2016; 11:e0152677.

34. Augustijns P, D'Hulst A, Van Daele J, Kinget R. Transport of artemisinin and sodium artesunate in Caco-2 intestinal epithelial cells. J Pharm Sci 1996; 85:577-579.

35. Crowe A, Ilett KF, Karunajeewa HA, Batty KT, Davis TM. Role of P glycoprotein in absorption of novel antimalarial drugs. Antimicrob Agents Chemother 2006; 50:3504-3506.
